# The Two Classes of Ceramide Synthases Play Different Roles in Plant Immunity and Cell Death

**DOI:** 10.3389/fpls.2022.824585

**Published:** 2022-04-07

**Authors:** Hong-Yun Zeng, He-Nan Bao, Yi-Li Chen, Ding-Kang Chen, Kun Zhang, Shuai-Kang Liu, La Yang, Yong-Kang Li, Nan Yao

**Affiliations:** State Key Laboratory of Biocontrol, Guangdong Provincial Key Laboratory of Plant Resource, School of Life Sciences, Sun Yat-sen University, Guangzhou, China

**Keywords:** *Arabidopsis thaliana*, cell death, ceramide synthases, immunity, salicylic acid, sphingolipid

## Abstract

Ceramide synthases (CSs) produce ceramides from long-chain bases (LCBs). However, how CSs regulate immunity and cell death in *Arabidopsis thaliana* remains unclear. Here, we decipher the roles of two classes of CS, CSI (LAG1 HOMOLOG 2, LOH2) and CSII (LOH1/3), in these processes. The *loh1-2* and *loh1-1 loh3-1* mutants were resistant to the bacterial pathogen *Pseudomonas syringae* pv *maculicola* (*Psm*) DG3 and exhibited programmed cell death (PCD), along with increased LCBs and ceramides, at later stages. In *loh1-2*, the *Psm* resistance, PCD, and sphingolipid accumulation were mostly suppressed by inactivation of the lipase-like proteins ENHANCED DISEASE SUSCEPTIBILITY 1 (EDS1) and PHYTOALEXIN DEFICIENT 4 (PAD4), and partly suppressed by loss of SALICYLIC ACID INDUCTION DEFICIENT 2 (SID2). The LOH1 inhibitor fumonisin B1 (FB1) triggered EDS1/PAD4-independent LCB accumulation, and EDS1/PAD4-dependent cell death, resistance to *Psm*, and C16 Cer accumulation. Loss of LOH2 enhances FB1-, and sphinganine-induced PCD, indicating that CSI negatively regulates the signaling triggered by CSII inhibition. Like Cer, LCBs mediate cell death and immunity signaling, partly through the EDS1/PAD4 pathway. Our results show that the two classes of ceramide synthases differentially regulate EDS1/PAD4-dependent PCD and immunity *via* subtle control of LCBs and Cers in Arabidopsis.

## Introduction

Free long-chain bases (LCBs), the simplest form of sphingolipids, are synthesized *de novo* from serine and palmitoylcoenzyme A. In Arabidopsis (*Arabidopsis thaliana*), LCBs can be bound to a fatty acid (FA) through an amide linkage to produce ceramides (Cers) by the three Cer synthases (CSs) LAG1 HOMOLOG 1–3 (LOH1–3). The different CSs determine the varied chemical compositions of Cers. LOH1 and LOH3 are classified as class II CS enzymes (CSII) and prefer very-long-chain acyl-CoA and trihydroxy LCB as substrates. LOH2 is classified as CSI and prefers palmitoyl-CoA and dihydroxy LCB as substrates (Markham et al., 2011; Luttgeharm et al., 2015, 2016; Supplementary Figure 1). In plants, Cers can be modified to form three other groups of sphingolipids: hydroxyceramides (hCers), glucosylceramides (GlcCers), and glycosylinositolphosphoceramides (GIPCs; Dunn et al., 2004).

Besides being the common backbone of all sphingolipids, free LCBs also act as second messengers in multiple processes, such as regulation of stomatal closure (Worrall et al., 2008; Guo et al., 2012), freezing tolerance (Huang et al., 2017), autophagy (Zheng et al., 2018), programmed cell death (PCD; Shi et al., 2007), and immunity (Magnin-Robert et al., 2015; Liu et al., 2019). PCD is essential for normal growth and development as well as responses to a variety of biotic and abiotic stresses in plants (Locato and De Gara, 2018). Plant sphingolipid-associated PCD shares common features with the hypersensitive response cell death (Berkey et al., 2012), a commonly observed form of PCD triggered by avirulent pathogen infection (Locato and De Gara, 2018).

Genetic studies have revealed the importance of different CSs. The strong insertion mutant alleles *loh1-2*, *loh2-1*, *loh2-2*, and *loh3-2*, and the knockdown insertion alleles *loh1-1* and *loh3-1* exhibit a normal phenotype under standard growth conditions (Markham et al., 2011). The *loh1-2 loh3-2* double mutant is embryo-lethal, but some *loh1-1 loh3-1* mutant seeds survive and germinate into plants that develop smaller leaves; these leaves also senesce early (Markham et al., 2011). When grown under short-day conditions for 8–10 weeks, *loh1-2* mutant plants spontaneously develop yellow lesions, which form suddenly (within a few days), starting with the oldest leaves (Ternes et al., 2011). In addition, 6-week-old *loh1-2* plants accumulate elevated levels of free trihydroxy sphingoids (another name for LCBs) as well as Cer and GlcCer species with C16 FA backbones, suggesting that higher amounts of free trihydroxy sphingoids or C16-Cer species trigger the spontaneous cell death phenotype observed in *loh1* mutants (Ternes et al., 2011). However, no obvious phenotype is observed in the *loh3* single mutants, possibly due to the lower expression level of *LOH3*, compared with that of *LOH1* (Ternes et al., 2011). Overexpression of *LOH2*, but not of *LOH1* or *LOH3*, also induces PCD (Luttgeharm et al., 2015). We recently discovered that the onset of cell death induced by *LOH2* overexpression requires ENHANCED DISEASE SUSCEPTIBILITY 1 (EDS1) and PHYTOALEXIN DEFICIENT 4 (PAD4; Zeng et al., 2021), which are crucial components in salicylic acid (SA) biosynthesis and regulation of cell death (Lapin et al., 2020).

Fumonisin B1 (FB1), a potent inhibitor of CSs, is a powerful tool to explore the multiple functions of sphingolipids in plants (Zeng et al., 2020). In Arabidopsis, LOH1 has the highest K_i_ for FB1 when compared with LOH2 and LOH3, reflecting its sensitivity to FB1 (Luttgeharm et al., 2016). FB1 triggers the rapid accumulation of LCBs, especially the two main LCBs sphinganine (d18:0) and phytosphingosine (t18:0; Shi et al., 2007; Tsegaye et al., 2007; Saucedo-Garcia et al., 2011; Yanagawa et al., 2017). Prolonged exposure to FB1 causes excessive 16:0-sphingolipid biosynthesis (Markham et al., 2011). The *loh2-1* and *loh2-2* mutants are sensitive to FB1 (Mase et al., 2013), and lines overexpressing *LOH2* or *LOH3* are resistant to FB1 (Luttgeharm et al., 2015). Moreover, FB1 treatment results in the accumulation of SA (Abbas et al., 1994; Zhang et al., 2015), and SA promotes FB1-induced cell death (Zhang et al., 2015). FB1-mediated PCD in protoplasts was reported to be dependent on PAD4 and SA (Asai et al., 2000).

Free LCBs (such as d18:0 and t18:0) induce PCD in Arabidopsis (Shi et al., 2007). Tobacco (*Nicotiana tabacum*) BY-2 cell cultures challenged with d18:0 enter a death process (Lachaud et al., 2010). Arabidopsis MITOGEN ACTIVATED PROTEIN KINASE 6 (MPK6) appears to transduce the d18:0-induced PCD signal (Saucedo-Garcia et al., 2011). In addition, LCBs contribute to plant resistance against bacterial pathogens. Indeed, *PATHOGENESIS RELATED-1* (*PR1*) is more highly expressed in *loh1* plants and FB1-treated wild-type plants than in untreated wild type (Ternes et al., 2011; Zhang et al., 2015). t18:0 LCBs accumulate upon *Pseudomonas syringae* pv. *tomato* DC3000 infection (Peer et al., 2010), and regulate plasmodesmata functions and cell-to-cell communication through PLASMODESMATA-LOCATED PROTEIN 5 (PDLP5) at plasmodesmata membranes (Liu et al., 2019). Hence, even though their roles in plant immunity are still largely unknown, CSs appear to be important in immunity and cell death, acting by tapping into the LCB pool for their substrates.

Here, we demonstrate that CSII prevents the activation of immunity and PCD by repressing EDS1/PAD4 signaling, while CSI negatively regulates the immunity and PCD induced by the loss of CSII function, possibly by removing over-accumulated LCBs.

## Materials and Methods

### Plant Materials and Growth Conditions

Two Arabidopsis (*Arabidopsis thaliana*) ecotypes, Columbia-0 (Col-0) and Wassilewskija (Ws), were used in this study. The *loh1-1* and *loh3-1* mutants were in the Ws background; all other mutants were in the Col-0 background. The *pad4-1*, *eds1-2*, *sid2-1*, *loh1-2*, *loh2-2*, *loh3-2*, and *loh1-1 loh3-1* mutants were described previously (Markham et al., 2011; Bi et al., 2014; Zeng et al., 2021). The stable transgenic lines *LOH2 A*, *pad4-1 LOH2 A*, *eds1-2 LOH2 A*, *sid2-1 LOH2 A*, and *eds1-2 EDS1pro:EDS1-GFP* were described previously (Zeng et al., 2021). The double mutants *loh1-2 loh2-2*, *loh1-2 pad4-1*, *loh1-2 eds1-2*, and *loh1-2 sid2-1* were generated by crossing the *loh1-2* mutant with the *loh2-2*, *pad4-1*, *eds1-2*, and *sid2-1* single mutants, respectively. Plants were grown at 22°C, 60% relative humidity, and 60 μE m^−2^ s^−1^ (FSL, LED T8 22W) under a 16-h light/8-h dark cycle on soil (Jiffy, Netherlands).

### Confocal Microscopy Observations

The stable transgenic line *eds1-2 EDS1pro:EDS1-GFP* was used to monitor green fluorescent protein (GFP) fluorescence. The leaves from 3-week-old seedlings after FB1 or LCB treatment were observed with a laser scanning confocal microscope (LSM-880, Carl Zeiss, Germany). The excitation/emission wavelengths were 488/500–530 nm for GFP. All images were obtained with the same laser parameters.

### Pathogen Inoculation and Detection

The culture and infection of virulent strain *Pseudomonas syringae* pv. *maculicola* ES4326 strain DG3 (*Psm*DG3) was performed as previously described (Guttman and Greenberg, 2001). Briefly, the third to fifth leaves of 3- or 4-week-old plants were infiltrated with *Psm*DG3 at an OD_600_ of 0.001 [1.08 × 10^7^ colony forming units (cfu)/ml] or 10 mM MgSO_4_ (mock). For bacterial growth assays, at least 30 plants per line were tested and 6–24 independent leaf disks collected at 0, 2, or 3 days post-inoculation (dpi). The leaf disks were ground in 200 μl 10 mM MgSO_4_, and serial dilutions were plated on King’s B medium (King et al., 1954) with 100 μg/ml streptomycin and 50 μg/ml kanamycin. Bacterial cfu were counted 2 days after incubation at 28°C.

### Chemical Treatments

**Figure 1:**
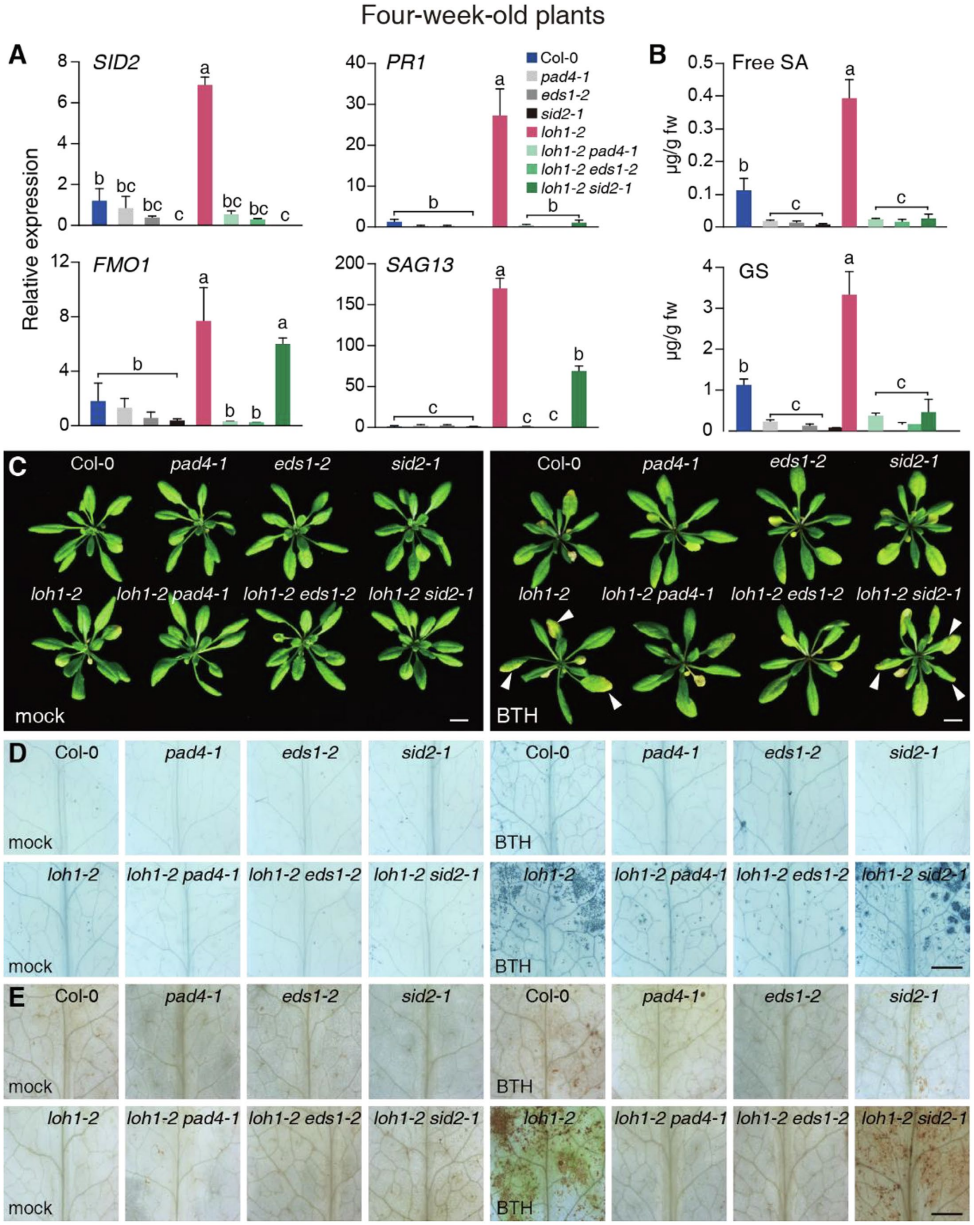
Effect of the *pad4-1, eds1-2, and sid2-1* mutations on benzothiadiazole (BTH) treatment in *loh1-2*. **(A)** Relative transcript levels of *SALICYLIC ACID INDUCTION DEFICIENT 2* (*SID2*), *PATHOGENESIS RELATED-1* (*PR1*), *FLAVIN-DEPENDENT MONOOXYGENASE 1* (*FMO1*), and *SENESCENCE-ASSOCIATED GENE 13* (*SAG13*) in 4-week-old plants. *ACT2* served as the internal control. Values were normalized to Col-0 levels (set to 1). **(B)** Free salicylic acid (SA) and glucosylated SA (GS) levels were measured in the indicated plants. **(C)** Representative images of plants (inflorescence stems were removed before we photographed the plants) treated with BTH. Three-week-old plants were sprayed with 300 μM BTH and observed 1 week later. White arrowheads indicate cell death lesions. Bar = 1 cm. **(D)** Trypan blue staining of leaves from **(C)**. Bar = 1 mm. **(E)** DAB staining of leaves from **(C)**. Bar = 1 mm. Values are means ± SE from triplicate biological repeats; different letters indicate significant differences, as determined by Fisher’s PLSD (*p* < 0.05) in **(A,B)**. At least, 40 plants per line were tested each time in **(C)** and at least 12 leaves per line were stained each time in **(D,E)**. All experiments were conducted at least three times using independent samples.

For benzothiadiazole (BTH) treatments, 3-week-old seedlings grown on soil were sprayed with 300 μM BTH (ECG4310, Wako) prepared from a 100-mM stock solution in acetone or with 0.3% acetone (for mock treatment). For salicylic acid (SA) treatment, 3-week-old seedlings grown on soil were sprayed with 200 μM SA (S7401, Sigma) prepared from a 200-mM stock solution in ethanol or with 0.1% ethanol (mock). Samples were collected at the indicated time points in Figures 1C–E and Supplementary Figure 5.

**Figure 2:**
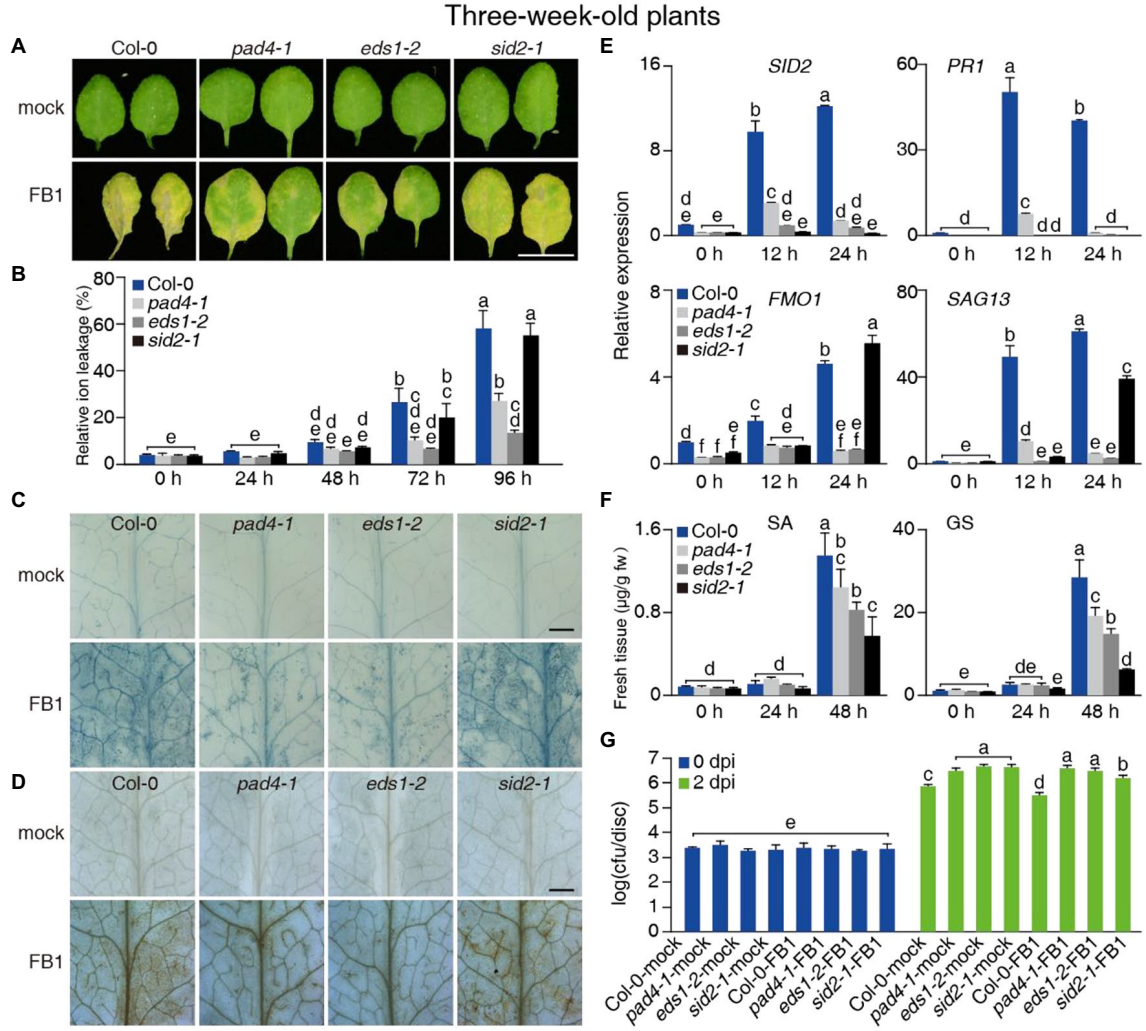
Effect of the *pad4-1*, *eds1-2*, and *sid2-1* mutations on FB1-triggered cell death and resistance to *Psm*DG3. **(A–F)** Three-week-old Col-0, *pad4-1*, *eds1-2*, and *sid2-1* plants were treated with mock or 10 μM FB1. **(A)** Representative images of detached leaves 4 days after treatment. Bar = 1 cm. **(B)** Relative ion leakage detection of plants 24–96 h after treatments. **(C,D)** Trypan blue **(C)** and DAB **(D)** staining of plants 3 days after treatments. Bars in **(C,D)** = 1 mm. **(E)** Relative transcript levels for *SID2*, *PR1*, *FMO1*, and *SAG13* in plants 12 and 24 h after 10 μM FB1 treatments. *ACT2* served as internal control. Values were normalized to Col-0 levels (set to 1). **(F)** Free SA and GS levels in plants 24 and 48 h after 10 μM FB1 treatments. **(G)** Growth of *Psm*DG3 in FB1-treated plants 2 dpi. The plants were sprayed with 0.5 mM FB1 1 day before *Psm*DG3 inoculation. Values are means ± SE from triplicate biological repeats in **(B,E,F)**, six biological replicates at 0 dpi and 24 biological replicates at 2 dpi in **(G)**. Different letters indicate significant differences assessed among all genotypes/time points, as determined by Fisher’s PLSD (*p* < 0.05; **B,E–G**). At least 30 plants per line were tested each time in **(A,B,G)**, at least 12 leaves per line were stained each time in **(C,D)**. All experiments were conducted at least three times independently.

**Figure 3:**
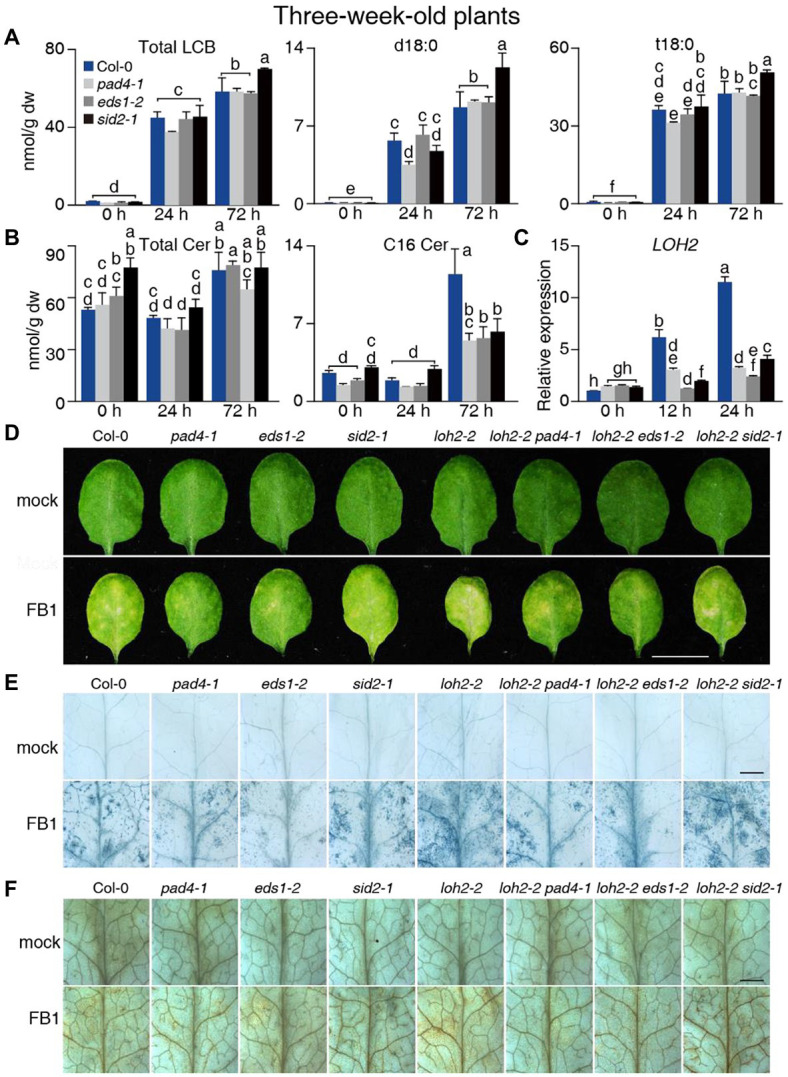
Sphingolipid profile of 3-week-old Col-0, *pad4-1-*, *eds1-2-*, and *sid2-1*-plants upon FB1 treatment. **(A)** Total contents of LCB, d18:0, and t18:0. **(B)** Total contents of Cer and C16 Cer. **(C)** Relative *LOH2* transcript levels in plants before (0 h), 12 or 24 h post 10 μM FB1 treatment. *ACT2* served as the internal control. Values were normalized to Col-0 levels (set to 1). **(D)** The phenotype of plants 2 days post-FB1 treatment. Bar = 1 cm. **(E)** Trypan blue staining of leaves from **(D)**. Bar = 1 mm. **(F)** DAB staining of leaves from **(D)**. Bar = 1 mm. Values are means ± SE from triplicate biological repeats in **(A–C)**. Different letters indicate significant differences between all genotypes/time points, as determined by Fisher’s PLSD (*p* < 0.05). At least 30 plants per line were tested each time in **(D)** and at least 12 leaves per line were stained each time in **(E,F)**. The experiments were repeated three times using independent samples.

**Figure 4:**
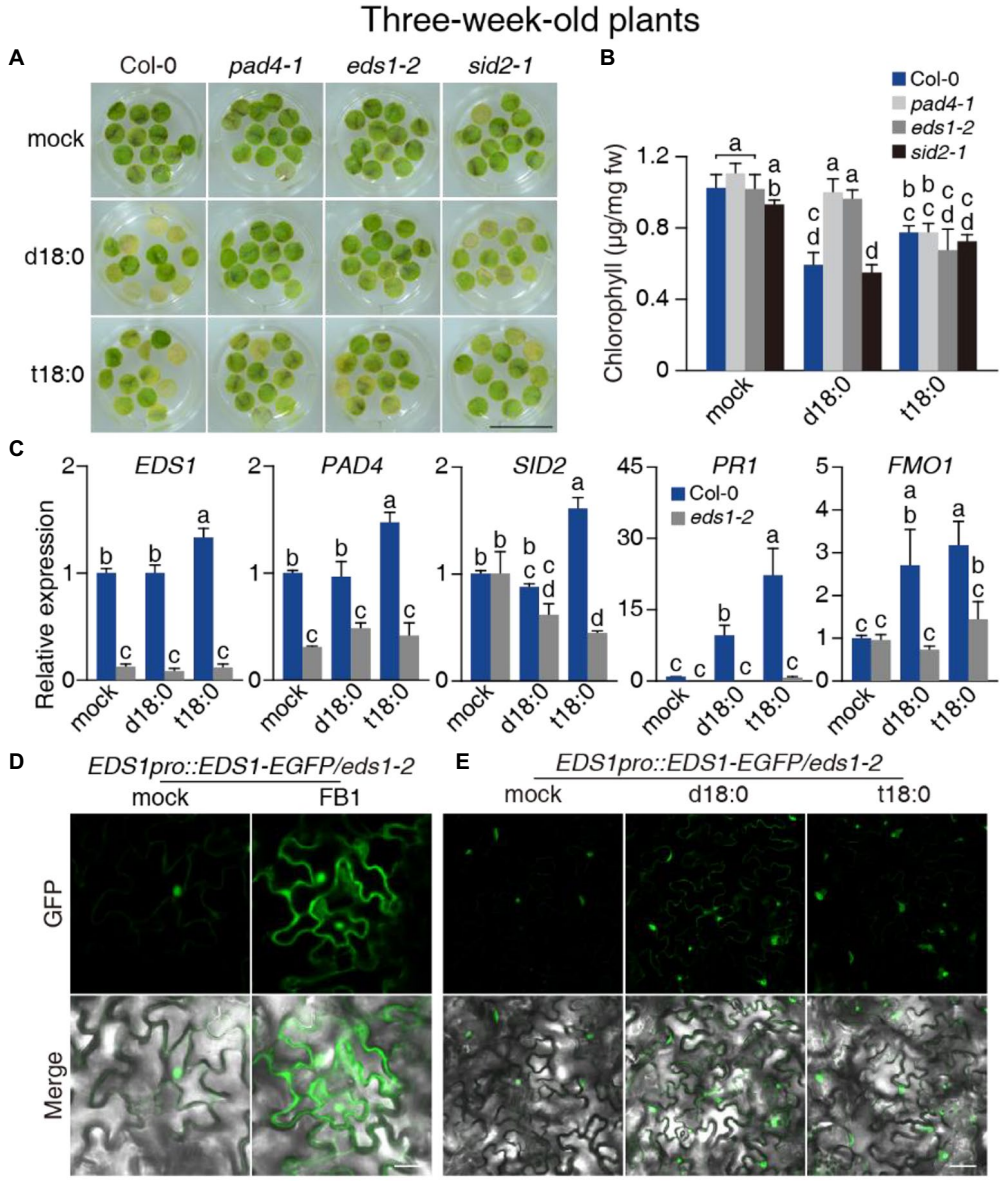
Effect of the *pad4-1*, *eds1-2*, and *sid2-1* mutations on LCB-induced cell death and immunity and the response of EDS1 protein to LCBs. **(A)** Representative images of detached leaf disks 5 days after 100 μM LCBs (d18:0 or t18:0) treatment. At least 36 leaf disks per line were test each time. Bar = 1 cm. **(B)** Chlorophyll content 5 days after treatment from **(A)**. **(C)** Relative transcript levels for *EDS1*, *PAD4*, *SID2*, *PR1*, and *FMO1* in 3-week-old plants 24 h after 0.5% ethanol (mock) treatment or treatment with 100 μM d18:0 or t18:0. *ACT2* served as the internal control. Values were normalized to Col-0 levels (set to 1). **(D,E)** EDS1 protein accumulated in FB1-treated **(D)** and LCB-treated **(E)** 3-week-old *eds1-2 EDS1pro:EDS1-EGFP* transgenic plants. A representative confocal live epidermal cell image was shown from at least 30 leaves per line. Bar = 20 μm. Values are means ± SE from triplicate biological repeats in **(B,C)**. Different letters indicate significant differences between all genotypes/treatments, as determined by Fisher’s PLSD (*p* < 0.05) in **(B,C)**. All experiments were conducted at least three times using independent samples.

For fumonisin B1 (FB1) treatment, the third to fifth leaves of 3-week-old plants grown on soil were infiltrated with 10 μM FB1 prepared from a 1 mM stock solution containing 10% methanol or with 0.1% methanol (mock). Samples were collected at the indicated time points in Figures 2A–F, 3, 4D and Supplementary Figures 9,10, 11A–C.

For treatment with LCB sphingolipids shown in Figures 4A,B, leaf disks were collected from the third to fifth leaves of 3-week-old plants grown on soil. The leaf discs were placed in the wells of 12-well plates containing 100 μM LCB (d18:0 or t18:0) prepared from a 20 mM stock solution in ethanol or 0.5% ethanol (mock). The 12-well plates were incubated in the greenhouse. In Figures 4C,E, the third to fifth leaves of 3-week-old plants grown on soil were infiltrated with 100 μM LCB (d18:0 or t18:0) or 0.5% methanol (mock). In Supplementary Figures 11D–I, the third to fifth leaves of 3-week-old plants grown on soil were infiltrated with 1 mM LCB (d18:0 or t18:0) or 0.1% methanol (mock).

For combined treatments with BTH and FB1, 3-week-old plants grown on soil were sprayed with 300 μM BTH or 0.3% acetone (mock), followed by infiltration of the third to fifth leaves of the same plants with 10 μM FB1 24 h later. Samples were collected at the indicated time points in Supplementary Figure 9.

For combined treatments with FB1 and *Psm*DG3, 3-week-old plants grown on soil were sprayed with 0.5 μM FB1 or 0.005% methanol (mock), followed by inoculation of the third to fifth leaves of the same plants with *Psm*DG3 at an OD_600_ of 0.001 24 h later. Samples were collected at the indicated the time points in Figure 2G.

### Trypan Blue Staining and DAB Staining

**Figure 5:**
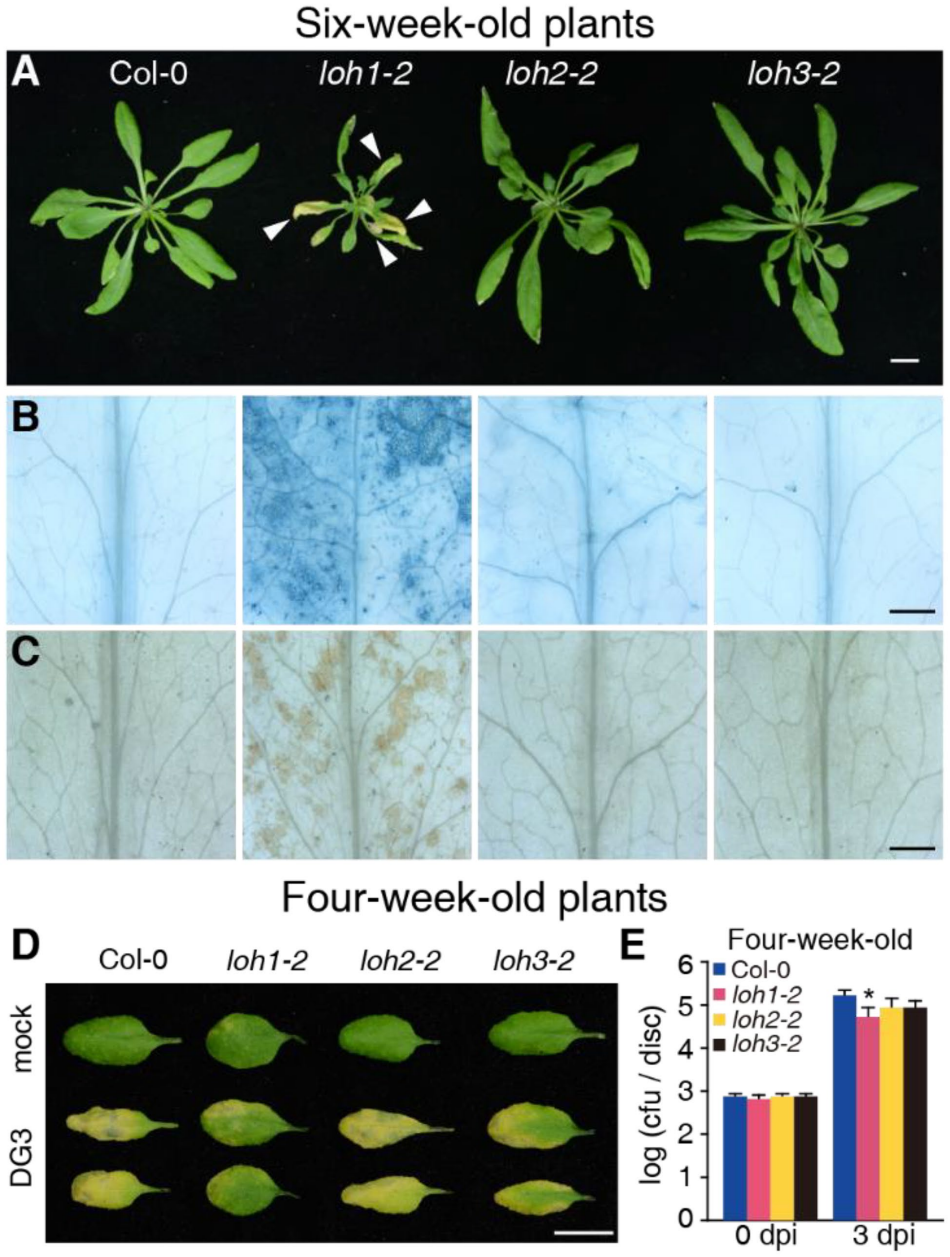
Defense responses of Col-0, *loh1-2*, *loh2-1*, and *loh3-2* plants. **(A)** Growth phenotypes of 6-week-old plants (inflorescence stems were removed before we photographed the plants). Bar = 1 cm. White arrowheads indicate cell death lesions. **(B)** Trypan blue staining of leaves from **(A)**. Bar = 1 mm. **(C)** 3,3′-diaminobenzidine (DAB) staining of leaves from **(A)**. Bar = 1 mm. **(D)** The rosette leaves from 4-week-old plants were inoculated with *Psm*DG3, and the pictures of leaves were taken 3 days after inoculation. Bar = 1 cm. **(E)** Growth of *Psm*DG3 in leaves from 4-week-old plants in **(D)**. Trypan blue **(B)** and DAB staining **(C)** of untreated leaves. At least 12 leaves per line were stained each time in **(B,C)**, and at least 30 plants per line were tested each time in **(D,E)**. Values are means ± SE from six biological replicates at 0 days post-infection (dpi) and 24 biological replicates at 3 dpi in **(E)**. Significant differences between Col-0 and *loh1-2*, *loh2-2*, or *loh3-2* were determined by Student’s *t*-test (^*^p < 0.05) in **(E)**. The experiments were conducted three times independently.

**Figure 6:**
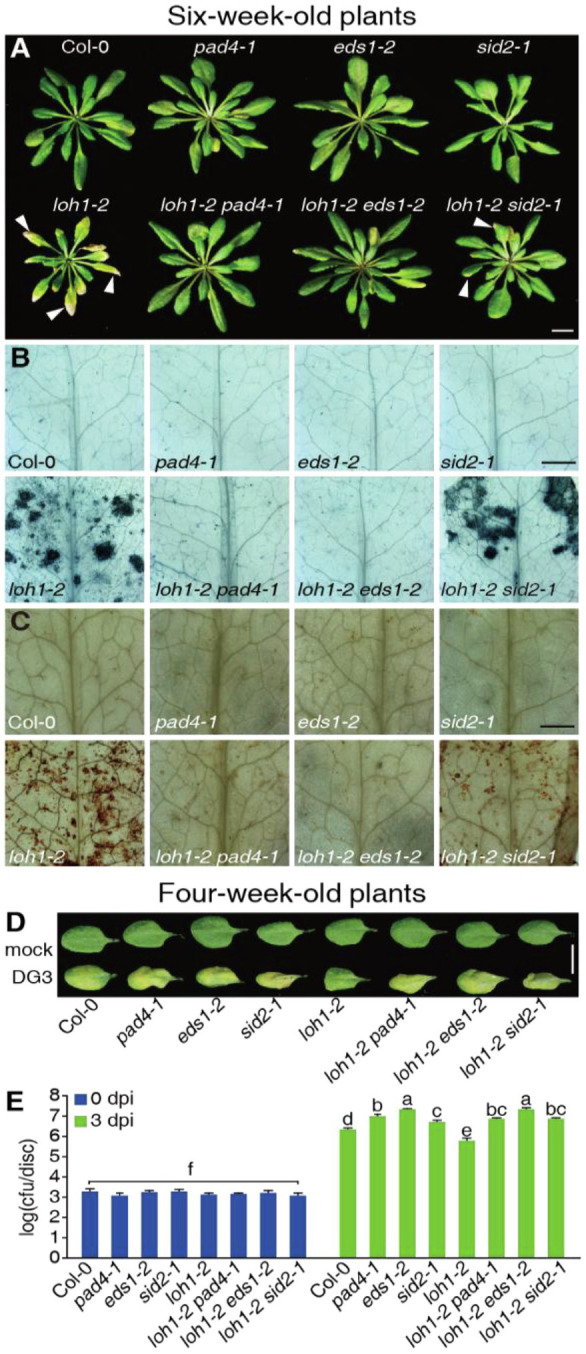
Effect of the *pad4-1*, *eds1-2*, and *sid2-1* mutations on the immune response and cell death caused by *loh1-2*. **(A)** Growth phenotypes of 6-week-old plants (inflorescence stems were removed before we photographed the plants). White arrowheads indicate cell death lesions. Bar = 1 cm. **(B)** Trypan blue staining of leaves from **(A)**. Bar = 1 mm. **(C)** DAB staining of leaves from **(A)**. Bar = 1 mm. **(D)** The rosette leaves from 4-week-old plants were inoculated with *Psm*DG3, and the pictures of leaves were taken 3 days after inoculation. Bar = 1 cm. **(E)** Growth of *Psm*DG3 in leaves from **(D)**. Trypan blue **(B)** and DAB staining **(C)** of untreated leaves. At least 12 leaves per line were stained each time in **(B,C)**. At least, 30 plants per line were tested each time in **(D,E)**. Values are means ± SE from six biological replicates at 0 dpi and 24 biological replicates at 3 dpi in **(E)**. Different letters indicate significant differences assessed among all genotypes/time points, as determined by Fisher’s protected least significant difference (PLSD; *p* < 0.05) in **(E)**. The experiments were conducted three times using independent samples.

Trypan blue staining and 3,3′-diaminobenzidine (DAB) staining were performed as described (Zeng et al., 2021). Briefly, the leaves were harvested at the indicated time points in Figures 1–3, 5, 6, and Supplementary Figures 2, 4, 5, 11 into solution containing 2.5 mg/ml trypan blue (Sigma-Aldrich), 25% (v/v) lactic acid, 25% (v/v) water-saturated phenol, 25% (v/v) glycerol, and 25% (v/v) distilled water, or into distilled water (pH 5.5) containing 1 mg/ml DAB (Sigma-Aldrich). The detached leaves were observed under a fluorescence microscope with a charge-coupled device (CCD) camera (AxioCam HRc; Carl Zeiss).

### Sphingolipid Assay

Samples for sphingolipid analysis were prepared and the components of sphingolipids were determined as described previously (Zeng et al., 2021). Briefly, sphingolipid was extracted from about 100 mg of leaves by the lower phase of isopropanol/hexane/water (55, 20, and 25, v/v/v) with internal standards (C17 base D-erythro-sphingosine, d18:1 C12:0-ceramide, d18:1 C12:0-glucosylceramide, and ganglioside GM1). Analyses of LCBs (where values for total LCBs are given, these are endogenous free LCBs), Cers, hCers, and GlcCers were performed using an ultrafast liquid chromatograph (UFLC LC-20A, Shimadzu), with a 2 × 150 mm Luna 3-μm column (Phenomenex) for C8 separation, coupled to a quadrupole time-of-flight (AB Sciex Triple TOF 5600). The raw mass spectrometry (MS) data (wiff.scan files) were collected with Analyst 1.6.1 software (Applied Biosystems) and peaks for individual sphingolipids were assigned based on elution time and fragments of known standards. Analyses of GIPCs were performed using a high-performance liquid chromatograph (HPLC 1200SL, Agilent) coupled to a Triple Quad (6410, Agilent) with a 2 × 150 mm Luna 3-μm column (Phenomenex) for C8 separation.

### Gene Expression Analysis by Quantitative PCR

Total RNA was extracted with a Plant RNA kit (R4151-02; Magen) and reverse-transcribed into first-strand cDNA using the Prime Script RT Reagent Kit (RR047A; Takara). Real-time PCR was performed using the SYBR Premix Ex TaqII kit (RR820A; Takara) on a LightCycler480 system (Roche) with the following conditions: initial denaturation at 95°C for 30 s, followed by 40 cycles of PCR (denaturing, 95°C for 5 s; annealing, 60°C for 30 s; extension, and 72°C for 20 s). Relative transcript levels were determined by applying the 2^-△△CT^ method and using *ACT2* as reference (Livak and Schmittgen, 2001). Primers used in this study are listed in Supplementary Table 6.

### Phytohormone Content Determination

Salicylic acid and glucosylated SA (GS) were extracted and measured by liquid chromatography–mass spectrometry (LC–MS) as described previously (Zeng et al., 2021). The powdered samples (50 mg) were sealed in microcentrifuge tubes containing 500 μl of extraction buffer (isopropanol: water: concentrated HCl; 2:1:0.002; v/v/v) with internal standards [10 ng D_4_-SA (OlChemim)]. The samples were analyzed by LC–MS (LC model no. UFLC-XR, Shimadzu) coupled with a quadrupole TripleTOF 5600+ System (AB SCIEX) using a Luna C18 column (150 × 2.1 mm, 2.6 μm, Phenomenex).

### Relative Electrolyte Leakage

Electrolyte leakage was determined as previously described (Yang et al., 2019). Briefly, the third to fifth leaves of 3-week-old rosettes were collected into 50-ml tubes at the indicated time points in Figure 2B and Supplementary Figure 9B. The conductivity of the solution was measured after adding 10 ml deionized water and gentle agitation at room temperature for 1 h. Afterward, the solution with the leaf was boiled in a boiling water bath for more than 10 min and cooled to room temperature before measuring total electrolyte strength. Relative electrolyte leakage was calculated by comparing the leaked ionic strength with the corresponding total ionic strength.

### Statistical Analyses

Results are expressed as means ± SE. Significant differences were determined by ANOVA *post-hoc* tests in conjunction with Fisher’s protected least significant difference (PLSD) test (*p* < 0.05) using different letters or determined by Student’s *t*-test (^*^p < 0.05, ^**^p < 0.05, and ns indicates no significance difference). The number of biological replicates is given in the legends.

### Accession Numbers

Sequence data from this article can be found in the Arabidopsis Genome Initiative or the GenBank/EMBL data libraries under accession numbers: At3g48090 (*EDS1*), At3g52430 (*PAD4*), At1g74710 (*SID2*), At1g19250 (*FMO1*), At2g29350 (*SAG13*), At2g14610 (*PR1*), At3g25540 (*LOH1*), At3g19260 (*LOH2*), At1g13580 (*LOH3*), and At3g18780 (*ACT2*).

## Results

### *loh1-2* Mutants Are Resistant to *Psm*DG3

We obtained the strong loss-of-function mutant alleles *loh1-2*, *loh2-2*, and *loh3-2* for phenotypic analysis. When grown under normal conditions for 3–4 weeks, *loh1-2*, *loh2-2*, and *loh3-2* mutant plants were indistinguishable from the wild-type Col-0 (Supplementary Figures 2A,D). We observed no cell death phenotype in 3-week-old *loh* mutant plants, based on trypan blue and diaminobenzidine staining, with some cell death in 4-week-old *loh1* plants (Supplementary Figure 2). Unexpectedly, *loh1-2* plants exhibited clear senescence and cell death phenotypes after growth for 6 weeks under normal conditions, while Col-0, *loh2-2*, and *loh3-2* plants did not (Figures 5A–C), although previous studies reported *loh1-2* only developed spontaneous cell death when grown under short-day conditions (Markham et al., 2011; Ternes et al., 2011).

We next investigated whether *LOHs* are required for plant immunity. To evaluate the potential involvement of LOHs during infection with *Psm*DG3, which was derived from *Psm* strain ES4326 (Liang et al., 2003), we measured relative *LOH* transcript levels in wild-type plants infected with *Psm*DG3. *LOH1* and *LOH2* expression was slightly upregulated 24 h after infection, while *LOH2* and *LOH3* expression strongly increased 48 h after infection (Supplementary Figure 3A). Following challenge of 4-week-old wild-type and mutant plants with *Psm*DG3, only the *loh1-2* mutant, but not *loh2-2* or *loh3-2*, was resistant to *Psm*DG3 infection relative to wild type (Figures 5D,E). Prior to *Psm*DG3 infection, the relative transcript levels of the defense-associated gene *PR1* were already higher in *loh1-2* compared with Col-0, *loh2-2*, and *loh3-2*, while *PR1* transcript levels rose to equally high levels in all genotypes following *Psm*DG3 infection (Supplementary Figure 3B), indicating that the resistance seen in *loh1-2* in response to *Psm*DG3 is associated with the pre-activation of defense-associated gene expression.

We also tested the weak but viable *loh1-1 loh3-1* double mutant for phenotyping. As previously reported (Markham et al., 2011), 5-week-old *loh1-1 loh3-1* mutants showed senescence and cell death phenotypes (Supplementary Figures 4A–C). Upon *Psm*DG3 infection, 3-week-old *loh1-1 loh3-1* plants were more resistant to the pathogen (Supplementary Figure 4D) and supported less bacterial growth (Supplementary Figure 4E) than the wild type. These results indicate that knocking down *LOH1* and *LOH3* triggers plant cell death and confers resistance to *Psm*DG3 infection.

### Defense Signaling Is Active in *loh1-2* Mutants

We recently showed that cell death induced by *LOH2* overexpression requires the crucial immune components EDS1 and PAD4 (Zeng et al., 2021). We therefore evaluated their potential involvement, as well as that of SID2, in the cell death and immune responses seen in *loh1-2* by generating double mutants. The *pad4-1* and *eds1-2* mutants mostly suppressed the cell death phenotype of *loh1-2* and the *sid2-1* mutant only partially suppressed this phenotype (Figures 6A–C). In agreement, the *pad4-1*, *eds1-2*, and *sid2-1* mutants largely abolished the resistance exhibited by the *loh1-2* mutant against *Psm*DG3 infection, as evidenced by the higher bacterial growth obtained in these double mutants (Figures 6D,E).

We next tested the transcript levels of defense-associated genes in wild-type and *loh* mutant plants. The expression of *SID2*, *PR1*, and *FLAVIN-DEPENDENT MONOOXYGENASE 1* (*FMO1*), a EDS1/PAD4-dependent and SA-independent gene (Cui et al., 2017), as well as the cell death marker *SENESCENCE-ASSOCIATED GENE 13* (*SAG13*) reached higher levels in the *loh1-2* single and *loh1-1 loh3-1* double mutant relative to their respective wild-type plants (Col-0 for *loh1-2* and Ws for *loh1-1 loh3-1*; Figure 1A; Supplementary Figure 4F). In addition, *SID2* and *PR1* transcript levels, which are regulated by EDS1/PAD4 and SA, were sharply lower in *loh1-2 pad4-1*, *loh1-2 eds1-2*, and *loh1-2 sid2-1* double mutants compared with wild type (Figure 1A). By contrast, transcript levels of *FMO1* and *SAG13* were much lower than wild type in *loh1-2 pad4-1* and *loh1-2 eds1-2* double mutants, but not in the *loh1-2 sid2-1* double mutant (Figure 1A), indicating that the cell death phenotype observed in *loh1-2* is EDS1/PAD4-dependent and partly SA-dependent. The *loh1-2* single mutant accumulated higher levels of SA and GS, but the *pad4-1*, *eds1-2*, or *sid2-1* mutants did not, either on their own or in combination with *loh1-2*, which was consistent with the *SID2* transcript levels measured in these mutants (Figures 1A,B).

We next asked whether the pre-activation of SA-signaling induced the cell death phenotype of *loh1-2*. Accordingly, we treated plants harboring the *loh1-2* mutation with SA or the SA-analog BTH. SA and BTH treatment resulted in cell death in *loh1-2*, but not in *loh2-2* or *loh3-2* (Supplementary Figure 5). Similarly, BTH induced cell death in the *loh1-2* single and the *loh1-2 sid2-1* double mutant; this effect was largely suppressed in the *pad4-1 loh1-2* and *eds1-2 loh1-2* double mutants (Figures 1C–E). These results indicated that SA promotes the development of cell death in *loh1-2* mutants.

### The Disruption of Sphingolipid Homeostasis in *loh1-2* Mutants Depends on EDS1 and PAD4

To investigate the roles of EDS1 and PAD4 in the regulation of sphingolipid metabolism during the initiation and execution of cell death in *loh1-2*, we used 3- and 6-week-old plants. The levels of total LCBs, Cer, hCer, and GlcCer in 3-week-old plants harboring the *loh1-2* mutation were comparable to the wild type (Supplementary Figure 6A). Individual LCBs and C16 Cer slightly accumulated over wild-type levels in *loh1-2* single or double mutant plants. We measured lower levels of C22, C24, and C26 Cer in *loh1-2* single and double mutant plants compared with the wild type (Supplementary Figures 6B,C). The contents of Cers with LCB moieties in *loh1-2* single and double mutant plants were similar to those of the wild type (Supplementary Figure 6D). We determined that most sphingolipid components do not change in the *loh1-2* single or double mutant plants, except for several VLCF-sphingolipids (Supplementary Table 1).

**Figure 7:**
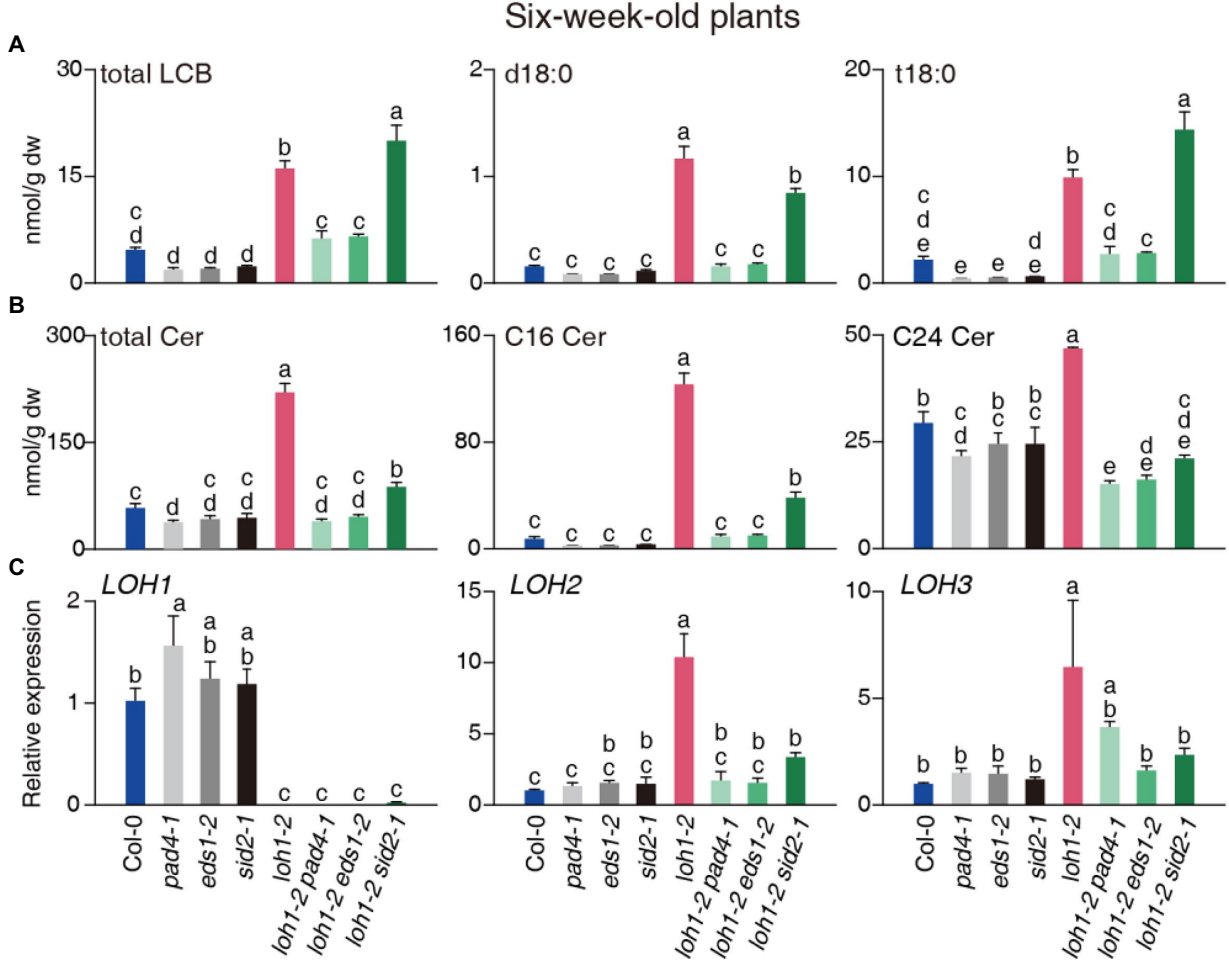
Total sphingolipid profile of 6-week-old Col-0, *pad4-1*, *eds1-2*, *sid2-1*, *loh1-2*, *loh1-2 pad4-1*, *loh1-2 eds1-2*, and *loh1-2 sid2-1* plants. Sphingolipids were extracted and detected by HPLC-ESI-TOF-MS/MS. **(A)** Total contents of total LCB, d18:0, and t18:0. **(B)** Total contents of total Cer, C16 Cer, and C24 Cer. **(C)** Relative transcript levels for *LOH1*, *LOH2*, and *LOH3* in uninfected plants. *ACT2* served as internal control. Values are means ± SE from triplicate biological repeats in **(A–C)**. Different letters indicate significant differences between genotypes, as determined by Fisher’s PLSD (*p* < 0.05). The experiments in **(A–C)** were conducted three times using independent samples.

By contrast, total LCBs, Cer, and hCer accumulated to high levels in 6-week-old *loh1-2*, but not in *pad4-1 loh1-2* and *eds1-2 loh1-2*, and to high or intermediate levels in *sid2-1 loh1-2*, while GlcCer and GIPC contents in *loh1-2*-single and double mutant plants were similar to wild type (Figures 7A,B; Supplementary Figure 7A). We detected high levels of LCBs, especially d18:0 and t18:0, in *loh1-2* and *loh1-2 sid2-1*, but not in *loh1-2 pad4-1* or *loh1-2 eds1-2* (Figure 7A; Supplementary Figure 7B). LCF Cer and VLCF Cer accumulated in the *loh1-2* single mutant, but their accumulation was mostly lost in *loh1-2 pad4-1*, *loh1-2 eds1-2*, and *loh1-2 sid2-1* plants (Figure 7B; Supplementary Figure 7B; Supplementary Table 2). The marked accumulation of Cer species with LCB moieties (d18:0 and t18:0 Cers) in *loh1-2* decreased in *loh1-2 pad4-1* and *loh1-2 eds1-2* double mutants (Supplementary Figure 7C; Supplementary Table 2).

We then asked whether the high levels of LCF Cer and VLCF Cer might be associated with LOH2 and LOH3 activity. First, we determined that *LOH2* and *LOH3* transcript levels are higher in the *loh1-2* single mutant relative to the wild type and double mutants (Figure 7C), indicating that loss of *LOH1* resulted in the induction of *LOH2* and *LOH3*. We also detected higher transcript levels for *LOH2* in the *loh1-1 loh3-1* double mutant in the Ws background (Supplementary Figure 4F). Since we observed accumulation of C16 Cer in *loh1-1*, we tested whether the high accumulation of LCF Cer seen in *LOH2*-overexpressing plants increased resistance to *Psm*DG3 infection. Indeed, *LOH2* overexpression (LOH2 A plants) enhanced plant resistance to *Psm*DG3 infection, but this effect was abrogated by the *pad4-1*, *eds1-2*, or *sid2-1* mutations (Supplementary Figures 8A,B), indicating that LCF Cer induces plant resistance through PAD4, EDS1, and SA.

### *eds1-2* and *pad4-1* Rescue Cell Death and Immunity Triggered by FB1

A previous study reported that FB1 inhibits LOH activity, especially LOH1 (Markham et al., 2011; Luttgeharm et al., 2016). Here, we asked whether PAD4 and EDS1 were required for plant responses to FB1. We infiltrated the leaves of 3-week-old wild-type, *pad4-1*, and *eds1-2* plants with FB1. As shown in Figure 2A, *pad4-1* and *eds1-2* infiltrated leaves were much greener than wild type at 4 days after FB1 treatment. In agreement with the leaf color, relative ion leakage was lower in *pad4-1* and *eds1-2* at 72 and 96 h after FB1 infiltration, compared with the wild type (Figure 2B). Trypan blue and DAB staining showed more limited cell death in FB1-treated *pad4-1* and *eds1-2* leaves, compared with treated wild-type leaves (Figures 2C,D).

To further elucidate the association between FB1 tolerance phenotypes seen in *pad4-1* and *eds1-2* mutants and the PAD4/EDS1-dependent signaling pathways, we analyzed the transcript levels of *SID2*, *PR1*, *FMO1*, and *SAG13* by RT-qPCR. The *eds1-2* and *pad4-1* single mutants significantly blocked the FB1-induced transcription of *SID2*, *FMO1*, and *PR1* at 12 and 24 h after FB1 treatment compared with wild-type plants (Figure 2E). We next measured endogenous SA levels in wild-type and mutant rosettes treated with FB1 for 24 or 48 h. Consistent with previous findings (Zhang et al., 2015), FB1 treatment induced SA levels in rosettes from the wild type and the *pad4-1* and *eds1-2* mutants relative to their respective controls after 48 h (Figure 2F). SA and GS levels were lower in *pad4-1* or *eds1-2* mutants and much lower in the *sid2-1* mutant compared with the wild type (Figure 2F). To investigate the role of SA in FB1-induced cell death, we pretreated plants with BTH 1 day before FB1 treatment. BTH treatment enhanced the FB1-sensitivity of wild-type and *sid2-1* mutant plants, but not of *pad4-1* or *eds1-2* mutant plants (Supplementary Figures 9A,B). These findings indicated that FB1 activates the SA pathway, partly through PAD4 and EDS1, to enhance cell death.

We asked whether FB1 also induced plant immunity *via* EDS1, PAD4, or SA in plants. To test this hypothesis, we pretreated 3-week-old plants with FB1 1 day before *Psm*DG3 inoculation. To largely avoid FB1-induced cell death, which would interfere with our tests, we used a lower concentration of FB1 (0.5 μM) rather than the one we used in other experiments (10 μM). Compared with controls, FB1 substantially induced resistance in Col-0 plants, but not in *pad4-1*, *eds1-2*, and *sid2-1* plants, as evidenced by the obtained cfu values, confirming the role of PAD4, EDS1, and SA in FB1-induced immunity (Figure 2G). Notably, combining this result with the observation that the FB1-induced cell death phenotype of *sid2-1* was not prominent, it seems that SA contributes more to FB1-induced immunity than FB1-induced cell death.

### *eds1-2* and *pad4-1* Rescued Cell Death in FB1-Treated *loh2-2* Mutants

We next detected and analyzed the changes in the sphingolipid profiles of wild-type and mutant plants after infiltration with FB1 for different periods. Total LCBs increased 26-fold in wild-type plants 24 and 72 h after FB1 treatment (Figure 3A), while we observed weak changes in Cer, hCer, or GlcCers levels (Figure 3B; Supplementary Figure 10B). Moreover, d18:0, d18:1, t18:0, and t18:1 content increased remarkably over controls, with t18:0 and d18:0 contributing the most in both the wild type and the mutants (Figure 3A; Supplementary Figure 10A). However, we detected little or no significant difference in the LCB content between wild-type and mutant plants for each time point (Figure 3A). Furthermore, the total Cer, C24 Cer, and C26 Cer contents increased slightly, while C16 Cer substantially increased in the wild type at 72 h post-treatment (Figure 3B; Supplementary Figure 10C). In addition, C16 Cer contents were much lower in the *pad4-1*, *eds1-2*, and *sid2-1* mutants compared with the wild type 72 h after FB1 treatment (Figure 3B). As with Cers, we detected higher levels of LCF hCer in the wild type compared with the mutants (Supplementary Tables 3**–**5). For Cer species with LCB moieties, the strong accumulation of d18:0 induced by FB1 treatment decreased by ~50% in *pad4-1*, *eds1-2*, and *sid2-1* mutants compared with the wild type (Supplementary Figure 10D; Supplementary Tables 3**–**5). Consistent with this, high levels of *LOH2* expression in the wild type were significantly suppressed in *pad4-1*, *eds1-2*, and *sid2-1* mutants (Figure 3C). These results aligned with our recent finding that LCF Cers accumulate in an EDS1/PAD4-dependent manner (Zeng et al., 2021).

We then investigated the role of LOH2 in FB1-induced cell death. The *loh2-2* plants exhibited stronger phenotypes after FB1 treatment than wild-type, *loh1-1*, and *loh3-2* plants (Supplementary Figures 11A–C). LOH2 likely prevents FB1-induced cell death by eliminating the effect of its substrate, d18:0. This hypothesis was confirmed by infiltrating wild-type and *loh2-2* plants with d18:0 (Supplementary Figures 11D–F). Indeed, *loh2-2* exhibited a more serious cell death phenotype than wild type after d18:0 treatment (Supplementary Figures 11D–F). Since LOH1 prefers t18:0 as a substrate, we also infiltrated the *loh1-2* mutant with t18:0. No obvious difference was observed in the mutant compared with wild type after t18:0 treatment (Supplementary Figures 11G–I).

### *eds1-2* and *pad4-1* Rescue Cell Death and Defense Gene Expression Induced by LCBs

To rule out any involvement of LOH2 in FB1-induced cell death, we crossed *loh2-2* to the *pad4-1*, *eds1-2*, and *sid2-1* mutants. When compared with wild type, all *loh2-2*-containing plants showed more serious cell death phenotypes. However, the *loh2-2 pad4-1* and *loh2-2 eds1-2* double mutants displayed fewer signs of cell death than the *loh2-2* single mutant (Figures 3D–F). This result implied that, besides preventing FB1-induced cell death through *LOH2* during the later period, EDS1 and PAD4 mainly promote LCB-induced cell death, since LCBs (d18:0 and t18:0) accumulated in FB1-treated plants.

We next exposed *pad4-1*, *eds1-2*, and *sid2-1* mutant plants to the d18:0 and t18:0 for phenotypic analysis. Since LCB treatments induced little cell death in wild-type plants (Supplementary Figures 11D–I), we treated detached leaf disks from the plants in 100 μM LCB rather than infiltrating the leaves with LCBs. As shown in Figure 4A, wild-type and *sid2-1* plants exhibited senescence phenotypes after treatment for 5 days. Unlike wild-type and *sid2-1* plants, the *pad4-1* and *eds1-2* plants remained green after treatment with d18:0 (Figure 4A). However, no significant difference was observed among the mutants after the t18:0 treatment (Figure 4B), indicating that EDS1 and PAD4 mainly regulate cell death triggered by d18:0, but not t18:0.

To investigate how LCBs mediate downstream signaling, we measured the transcript levels of EDS1 and PAD4-regulated genes upon d18:0 and t18:0 treatments. After d18:0 treatment for 24 h, only the expression levels of *PR1* and *FMO1* were elevated in wild type (Figure 4C). In the mutant plants treated with t18:0, besides *PR1* and *FMO1*, expression of *EDS1*, *PAD4*, and *SID2* were also induced (Figure 4C). The induction of these genes by d18:0 or t18:0 was largely compromised in *eds1-2* mutants (Figure 4C), suggesting that both d18:0- and t18:0-induced immunity required the EDS1 signaling pathway. To test whether LCBs regulated EDS1 turnover as well, we made stable transgenic plants expressing *EDS1-GFP* in the *eds1-2* mutant background (*eds1-2 EDS1pro:EDS1-GFP*) for FB1 and LCB treatments. As shown in Figures 4D,E, EDS1 protein accumulated in both the nucleus and the cytoplasm upon treatment with FB1 or LCBs, indicating that LCBs mediate downstream signaling in both the nucleus and the cytoplasm.

## Discussion

**Figure 8:**
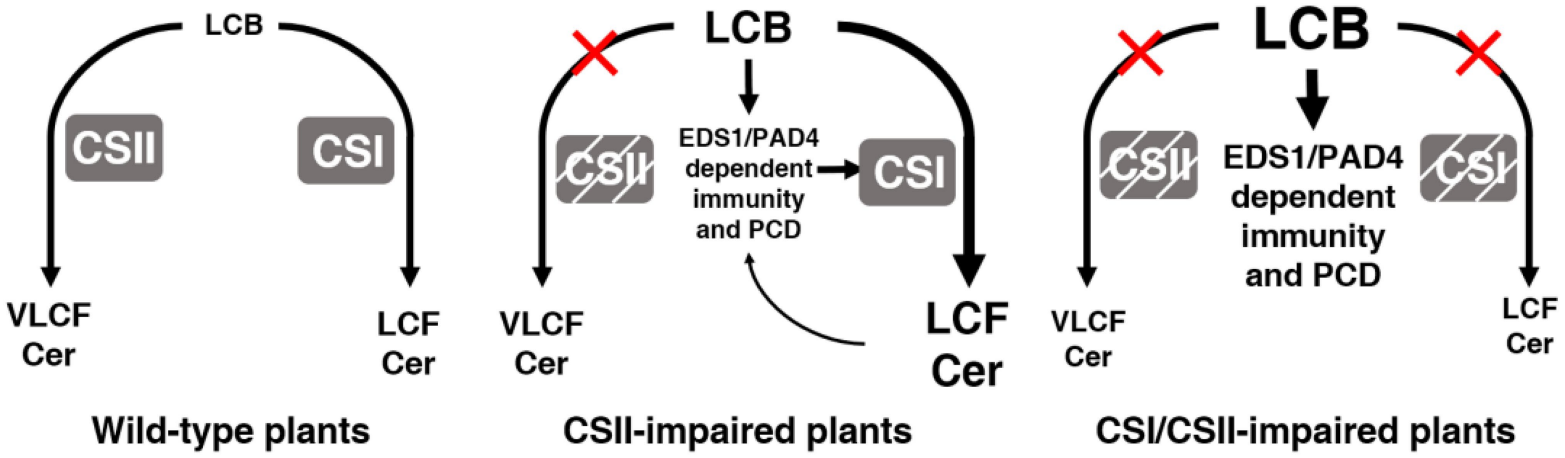
The roles of Cers in plant immunity and cell death. Model summarizing the roles of CSI and CSII in immunity and cell death. In wild type, normal levels of LCBs are maintained by the action of CSI and CSII. With the loss of CSII, LCBs accumulate, and EDS1/PAD4 act downstream to trigger immunity and cell death, while CSI partially compensates for the loss of CSII by consuming LCBs. With the loss of both CSI and CSII, higher levels of LCBs result in stronger phenotypes.

The precise control of LCBs and Cers is crucial for plants to survive and balance immunity in the face of pathogen attacks. Disruption of LCB/Cer homeostasis often causes growth inhibition, PCD, or activation of immune responses in plants (Zeng et al., 2020). CSs regulate the balance between LCBs and Cers, primarily by consuming LCB to synthesize Cer. Arabidopsis has two kinds of CSs that produce Cers with varied chemical compositions (Markham et al., 2011; Luttgeharm et al., 2016). However, how these two classes of CSs differentially regulate PCD, as well as immunity, through LCB and Cer metabolism in Arabidopsis remains largely unknown. Here, we characterize the contribution of various CSs to LCB levels and plant defense against a virulent pathogen. In wild type (Figure 8, left panel), CSI and CSII enzymes maintain normal levels of LCBs. In the absence of CSII (Figure 8, middle panel), LCBs accumulate, resulting in the activation of immunity and cell death *via* EDS1/PAD4. In this case, CSI partially compensates for the loss of CSII by using the accumulating LCBs as substrates. However, when both CSI and CSII are missing (Figure 8, right panel), higher levels of LCBs accumulate, leading to stronger phenotypes. These findings contribute to our understanding of the complex network behind Cer biosynthesis that determines plant cell fate and immunity.

LAG1 HOMOLOG 1 and LOH3 (CSII) and LOH2 (CSII) appear to exert opposite effects on growth and cell death regulation (Markham et al., 2011; Ternes et al., 2011; Luttgeharm et al., 2015). We found that loss of LOH1 triggers the accumulation of LCBs, resulting in EDS1-, PAD4-, and SA-dependent cell death and immunity, but overexpression of LOH2 promotes the accumulation of C16 Cers, initiating EDS1-, PAD4-, and SA-dependent cell death and immunity. In this study, we discovered resistance to a virulent strain of *Psm* and cell death phenotypes at later stages of growth under our growth conditions for strong *loh1* mutants and the weak *loh1-1 loh3-1* double mutants (Figure 5; Supplementary Figures 2, 4). Plants from the strong *loh1-2* allele were reported to exhibit no obvious visible phenotypes when grown in long-day conditions (Markham et al., 2011), and only showed early senescence under short-day conditions (Ternes et al., 2011). One possible reason is that the *loh1-2* phenotype is sensitive to environmental conditions. Likewise, the *neutral ceramidase 1* (*ncer1*) T-DNA insertion mutant was first reported to have no visible phenotypes, although it accumulates hCers (Li et al., 2015), but was later described to exhibit developmentally controlled cell death (Zienkiewicz et al., 2019). Another sphingolipid-related mutant, *accelerated cell death 5* (*acd5*), also shows phenotypes that are modulated by the environment. For example, the *acd5* phenotypes depend on the age of the plants (Bi et al., 2014) and are enhanced by SA but suppressed by abscisic acid (ABA) and sodium chloride treatment (Yang et al., 2019). The fact that SA or BTH induces cell death in the *loh1-2* mutant background (Figure 1; Supplementary Figure 5) suggests that SA affects the initiation of cell death in *loh1-2*, as it does in *acd5*. Indeed, cultivation conditions influence the phenotypes displayed by the SA mutant collection (Pluharova et al., 2019).

The accumulation of sphingolipids in the *loh1-2* mutant (Figure 7; Supplementary Figures 6,7), accompanied by a cell death phenotype, was also reminiscent of the *acd5* mutant (Bi et al., 2014). However, unlike *acd5*, which has a similar sphingolipid profile to wild type at the early growth stage, *loh1-2* mutant seedlings contained less C24 Cers than the wild type (Supplementary Figure 6; Supplementary Table 1), confirming the role of LOH1 in the biosynthesis of VLCF Cers. Interestingly, LCBs accumulated to normal levels in young *loh1-2* seedlings (Supplementary Figure 6), indicating that the cell death phenotype in the mutant is triggered by high LCB levels, which accumulate later in development (Figure 7). Older *loh1-2 pad4-1* and *loh1-2 eds1-2* double mutant plants shared sphingolipid profiles similar to those of the wild type (Figure 7), suggesting that EDS1 and PAD4 control the initiation of cell death or the accumulation of LCBs. This is supported by a recent finding that deletion of *EDS1* or *SID2* reduces LCB contents in *FA hydroxylase* (*fah1*, *fah2*) and *loh2* triple mutants, even though the exact mechanism is still unknown (König et al., 2021). However, this hypothesis cannot explain why the *pad4-1*, *eds1-2*, and *sid2-1* mutants show almost no effect on the LCB accumulation induced by FB1 treatment (Figure 3; Supplementary Figure 10; Supplementary Tables 3**–**5). We hypothesize that in *loh1-2* mutant plants, LCB levels are kept in check when EDS1 or PAD4 is absent, while FB1-treated plants experience a rise in LCB levels that is too fast to mitigate. In support of this idea, the *sid2-1* mutant minimized the rise of LCB levels in the *loh1-2* background but not when exposed to FB1 (Figures 3A, 7A).

The lack of LCF Cer in *loh2-1* mutants did not result in visible developmental phenotypes under normal growth conditions (Markham et al., 2011; Ternes et al., 2011; Luttgeharm et al., 2015; Figures 5A–C; Supplementary Figure 2), or in a resistant phenotype upon infection by *Psm*DG3 (Figures 5D,E), indicating that maintaining normal levels of LCF Cer is not essential for plant growth or pathogen resistance. This may be due to the low concentration of d18:0 and d18:1, the main substrates of LCF Cer (Luttgeharm et al., 2015). However, high levels of LCF Cer induce cell death (Zeng et al., 2021) and enhance plant immunity against *Psm*DG3 through EDS1, PAD4, and SA (Supplementary Figure 8). Therefore, both the resistance and cell death phenotypes of *loh1-2* or *LOH2*-overexpression lines are associated with EDS1 and PAD4 signaling.

Long-chain base biosynthesis is regulated by the serine palmitoyl-CoA transferase complex and its regulators, orosomucoid-like proteins (Gonzalez-Solis et al., 2020). The availability of LCBs from CS activities also controls LCB homeostasis. The cell death phenotype seen in *loh1* mutants or upon treatment with FB1 appears to be due to the extremely high accumulation of LCBs (Shi et al., 2007; Markham et al., 2011; Ternes et al., 2011), since FB1-triggered cell death can be effectively impaired by disrupting the serine palmitoyl-CoA transferase complex (Zeng et al., 2020) and LCBs induce PCD (Shi et al., 2007; Saucedo-Garcia et al., 2011). However, the contribution of LCF Cer to cell death cannot be completely excluded in these cases, since LCF Cers also accumulate in *loh1* mutants and plants subjected to long-term FB1 exposure (Markham et al., 2011; Ternes et al., 2011).

Based on the observation that the rise in LCF Cer species 3 days after FB1 treatment was suppressed in the *pad4-1*, *eds1-2*, and *sid2-1* mutants (Figures 3A–C), we speculate that EDS1, PAD4, and SA promote LCF Cer accumulation to relieve high LCB toxicity to a certain extent during the later period of FB1-elicited cell death. This is consistent with the observations that *LOH2*-overexpressing plants are more resistant to FB1 (Luttgeharm et al., 2015) despite showing a cell death phenotype in normal conditions, and that loss of LOH2 aggravates FB1- and d18:0-induced cell death (Mase et al., 2013; Figure 3; Supplementary Figure 11). Notably, pre-activation of the SA pathway significantly enhances FB1-induced cell death (Supplementary Figure 9). In line with the dual roles of SA in FB1-induced cell death, *sid2* mutants shared similar cell death phenotypes with wild type during the later period following FB1 treatment (Figures 2A,B). SA has well-known dual roles in effector-triggered cell death (Zavaliev et al., 2020), and it will be very interesting to investigate whether a high level of SA alters sphingolipid metabolism to affect the outcome of effector-triggered cell death.

Several stress response proteins, such as MPK6 and PDLP5, were reported to bind to LCB to trigger cell death or immunity (Saucedo-Garcia et al., 2011; Liu et al., 2019). Regulation of enzyme activity by LCBs was observed previously, as with the calcium-dependent kinase CPK3, which can be activated by d18:0, leading to cell death in plants (Lachaud et al., 2013). However, no protein has been reported to regulate both LCB- and Cer-induced cell death until now. EDS1 was reported to participate in the induction of cell death in response to both abiotic and biotic stress (Rustérucci et al., 2001; Ochsenbein et al., 2006; Straus et al., 2010; Wituszynska et al., 2015). We discovered here that, like C16 Cer (Zeng et al., 2021), LCBs regulate the signaling cascade downstream of EDS1 and PAD4. In addition, treatment with FB1, d18:0, or t18:0 elevates EDS1 protein levels in the nucleus and the cytoplasm (Figure 4), possibly leading to transcriptional activation of downstream genes *via* nucleus-localized EDS1 while accelerating senescence and cell death *via* cytoplasm-localized EDS1. Whether LCBs directly or indirectly regulate EDS1 and/or PAD4 to induce immunity and cell death remains unknown. As we showed in Figure 4, d18:0 and t18:0 LCBs act differently in EDS1/PAD4-regulated cell death and immunity. It will be very interesting to pursue the molecular mechanisms underlying the distinct functions of d18:0 and t18:0.

In this study, we focused on the roles of LOHs in plant defense against a virulent pathogen, *Psm*DG3. SA and EDS1 signaling, which have well-known functions in basic immunity (Lapin et al., 2020), are involved in the defense response that occurs due to loss of *LOH1*. Alteration of LCBs or Cers induces PCD in plants (Berkey et al., 2012; Ali et al., 2018; Huby et al., 2020). One important question is whether LCBs or Cers engage in the hypersensitive response (HR) or HR cell death in plants. However, we lack evidence that sphingolipid-associated cell death is a form of HR cell death. Moreover, the role of sphingolipids in effector-triggered immunity (ETI)-associated HR is also unclear. Infection with avirulent bacteria, such as *Pst* (*avr*Rpm1), triggers *de novo* synthesis of t18:0 from d18:0 in Arabidopsis (Peer et al., 2010). However, application of t18:0 LCB produced little or no effect on *avr*Rpm1-triggered cell death (Glenz et al., 2019). Interestingly, d18:0 strongly reduced HR and reactive oxygen species production upon challenge with *Pst* AvrRPM1 (Magnin-Robert et al., 2015). Inoculation with *PsmES4326* carrying *avrRpt2* or *avrRpm1* elicited the normal HR in the ceramide-accumulation mutant *acd5* plants, compared with that in wild type (Greenberg et al., 2000), indicating that Cers may also not be essential for ETI. Hence, the PCD-promoting function of LCBs and Cers seem to be negligible in rapid ETI–HR during plant–pathogen interactions. Instead, moderate accumulation of LCBs or Cers may mainly induce plant defense responses through SA and EDS1 signaling without causing PCD. When the accumulation of LCBs or Cers is out of control, plant overactivate SA and EDS1 signaling, leading to PCD without pathogen infection. Since high levels of SA inhibited HR cell death and simultaneous accumulation of nuclear and cytoplasmic EDS1 do not trigger cell death in ETI (Garcia et al., 2010; Zavaliev et al., 2020), alteration of LCBs or Cers may repress HR cell death rather than enhance it. Therefore, it seems that alteration of LCBs and Cers could mainly induce basal resistance, although more evidence is need to test this hypothesis.

## Author’s Note

The author responsible for distribution of materials integral to the findings presented in this article in accordance with the policy described in the Instructions for Authors is: NY (yaonan@mail.sysu.edu.cn).

## Data Availability Statement

The datasets presented in this study can be found in online repositories. The names of the repository/repositories and accession number(s) can be found in the article/**Supplementary Material**.

## Author Contributions

H-YZ and NY conceived and designed the experiments, analyzed the data, and wrote the manuscript. H-YZ, H-NB, Y-LC, D-KC, KZ, S-KL, LY, and Y-KL performed the experiments. NY contributed to reagents, materials, and analysis tools. All authors contributed to the article and approved the submitted version.

## Funding

This work was supported by the National Natural Science Foundation of China (32070196 and 31771357), the Natural Science Foundation of Guangdong Province (2019B1515120088 and 2017A030311005), and Sun Yat-sen University (Project 33000-31143406).

## Conflict of Interest

The authors declare that the research was conducted in the absence of any commercial or financial relationships that could be construed as a potential conflict of interest.

## Publisher’s Note

All claims expressed in this article are solely those of the authors and do not necessarily represent those of their affiliated organizations, or those of the publisher, the editors and the reviewers. Any product that may be evaluated in this article, or claim that may be made by its manufacturer, is not guaranteed or endorsed by the publisher.
