# Supplementary material for: The Two Classes of Ceramide Synthases Play Different Roles in Plant Immunity and Cell Death
Source: Front Plant Sci. 2022 Apr 7;13:824585. doi: 10.3389/fpls.2022.824585 (PMC9021646; doi:10.3389/fpls.2022.824585)
Supplement: Supplementary file 1 [file Data_Sheet_1.docx]

**Supplemental Tables**

**Supplemental Table 1.** Sphingolipid profiles of 3-week-old WT, *pad4-1*, *eds1-2*, *sid2-1*, *loh1-2*, *loh1-2 pad4-1*, *loh1-2 eds1-2* and *loh1-2 sid2-1* leaves.

| **Species** | **WT** | ***pad4-1*** | ***eds1-2*** | ***sid2-1*** | ***loh1-2*** | ***loh1-2 pad4-1*** | ***loh1-2 eds1-2*** | ***loh1-2 sid2-1*** |
| --- | --- | --- | --- | --- | --- | --- | --- | --- |
| t18:0 LCB | 1.19±  0.11 | 0.8±  0.09 | 0.67±  0.12 | 1.18±  0.11 | 1.84±  0.32 | 2.99±  0.29 | 3.54±  0.34 | 4.9±  1.01 |
| t18:1 LCB | 2.54±  0.15 | 1.43±  0.37 | 1.3±  0.17 | 1.97±  0.21 | 2.95±  0.39 | 2.77±  0.18 | 2.92±  0.22 | 3.14±  0.32 |
| d18:1 LCB | 0.04±  0 | 0.04±  0 | 0.05±  0.01 | 0.06±  0 | 0.06±  0 | 0.02±  0 | 0.04±  0 | 0.04±  0 |
| d18:0 LCB | 0.2±  0 | 0.24±  0.08 | 0.15±  0 | 0.25±  0.04 | 0.2±  0.03 | 0.32±  0.02 | 0.41±  0.08 | 0.4±  0.05 |
| t18:0 c16:0 Cer | 1.2±  0.3 | 0.35±  0.05 | 0.27±  0.01 | 0.6±  0.15 | 1.34±  0.33 | 2.44±  0.12 | 3.05±  0.53 | 3.46±  0.98 |
| t18:0 c20:1 Cer | 0.1±  0 | 0.06±  0.01 | 0.06±  0 | 0.1±  0 | 0.07±  0 | 0.06±  0.01 | 0.06±  0.01 | 0.09±  0 |
| t18:0 c22:0 Cer | 1.36±  0.29 | 0.49±  0.13 | 0.5±  0.08 | 0.9±  0.23 | 0.88±  0.14 | 0.65±  0.08 | 0.7±  0.17 | 0.78±  0.33 |
| t18:0 c24:0 Cer | 0.33±  0.05 | 0.19±  0.07 | 0.24±  0.08 | 0.32±  0.05 | 0.61±  0.24 | 0.11±  0.01 | 0.09±  0.02 | 0.12±  0.04 |
| t18:0 c24:1 Cer | 5.21±  0.62 | 0.59±  0.45 | 1.6±  0.79 | 0.19±  0.05 | 1.78±  0.91 | 1.77±  0.16 | 1.7±  0.36 | 1.76±  1.23 |
| t18:0 c26:0 Cer | 1.32±  0.26 | 0.38±  0.12 | 0.65±  0.31 | 0.12±  0.01 | 0.76±  0.35 | 0.69±  0.06 | 0.73±  0.14 | 1.11±  0.46 |
| t18:0 c26:1 Cer | 0.09±  0 | 0.05±  0.01 | 0.06±  0.01 | 0.17±  0.09 | 0.1±  0.01 | 0.06±  0.01 | 0.05±  0.01 | 0.05±  0.01 |
| t18:1 c16:0 Cer | 3.16±  0.27 | 1.25±  0.15 | 1.34±  0.08 | 1.78±  0.18 | 2.83±  0.59 | 3.14±  0.3 | 4.14±  0.61 | 4.08±  0.49 |
| t18:1 c20:0 Cer | 0.16±  0 | 0.08±  0.01 | 0.08±  0.01 | 0.13±  0.02 | 0.09±  0.01 | 0.11±  0 | 0.11±  0.01 | 0.13±  0.03 |
| t18:1 c22:0 Cer | 1.29±  0.03 | 0.56±  0.14 | 0.55±  0.15 | 0.88±  0.22 | 0.6±  0.12 | 0.36±  0.05 | 0.38±  0.08 | 0.44±  0.2 |
| t18:1 c24:0 Cer | 13.08±1.17 | 7.62±  2.7 | 10.68±2.94 | 12.43±  1.75 | 7.79±  1.35 | 3.98±  0.27 | 3.54±  0.82 | 5.08±  2.11 |
| t18:1 c24:1 Cer | 8.64±  0.12 | 5.62±  1.71 | 6.06±  1.63 | 8.26±  1.78 | 3.7±  0.58 | 2.11±  0.23 | 1.79±  0.35 | 2.24±  0.85 |
| t18:1 c26:0 Cer | 6.46±  1.16 | 3.79±  1.34 | 6.01±  1.53 | 6.23±  0.95 | 3.36±  0.52 | 3.05±  0.12 | 2.87±  0.69 | 4.1±  1.55 |
| t18:1 c26:1 Cer | 1.31±  0.18 | 0.01±  0 | 0.01±  0 | 0.54±  0.53 | 0.41±  0.2 | 0.66±  0.05 | 0.56±  0.11 | 0.02±  0 |
| d18:0 c16:0 Cer | 0.29±  0.03 | 0.17±  0.05 | 0.1±  0 | 0.25±  0.05 | 0.25±  0.05 | 0.35±  0.01 | 0.39±  0.06 | 0.5±  0.14 |
| d18:1 c16:0 Cer | 0.19±  0.01 | 0.13±  0.03 | 0.1±  0.01 | 0.22±  0.03 | 0.14±  0.01 | 0.16±  0 | 0.15±  0.01 | 0.21±  0.03 |
| d18:1 c18:0 Cer | 0.09±  0.01 | 0.08±  0.01 | 0.12±  0.01 | 0.12±  0.02 | 0.11±  0 | 0.1±  0 | 0.13±  0.02 | 0.11±  0 |
| d18:1 c20:0 Cer | 0.06±  0 | 0.06±  0.01 | 0.06±  0 | 0.06±  0 | 0.06±  0 | 0.05±  0 | 0.08±  0.02 | 0.06±  0 |
| d18:1 c24:0 Cer | 0.28±  0.01 | 0.3±  0.04 | 0.34±  0.02 | 0.37±  0.06 | 0.29±  0.02 | 0.34±  0.03 | 0.39±  0.05 | 0.41±  0.01 |
| d18:1 c26:0 Cer | 0.03±  0 | 0.01±  0 | 0.03±  0 | 0.03±  0 | 0.02±  0 | 0.2±  0.01 | 0.24±  0.03 | 0.02±  0 |
| t18:0 h16:0 hCer | 0.3±  0.06 | 0.14±  0.03 | 0.11±  0.02 | 0.2±  0.05 | 0.33±  0.03 | 0.57±  0.12 | 0.48±  0.09 | 0.82±  0.45 |
| t18:0 h20:0 hCer | 0.02±  0.02 | 0.02±  0.01 | 0.01±  0 | 0±  0 | 0.01±  0.01 | 0.07±  0.01 | 0.03±  0.01 | 0.01±  0.01 |
| t18:0 h22:0 hCer | 0.45±  0.05 | 0.22±  0.06 | 0.14±  0.04 | 0.31±  0.1 | 0.45±  0.02 | 0.27±  0.09 | 0.21±  0.05 | 0.23±  0.1 |
| t18:0 h24:0 hCer | 3.89±  0.57 | 3±  0.94 | 3.36±  0.91 | 3.67±  1.02 | 4.43±  0.32 | 3.6±  0.81 | 2.92±  0.68 | 3.9±  1.41 |
| t18:0 h24:1 hCer | 1.34±  1.06 | 0.14±  0.03 | 0.14±  0.03 | 0.23±  0.05 | 1.83±  0.91 | 1.19±  0.08 | 1.3±  0.26 | 0.06±  0.02 |
| t18:0 h26:0 hCer | 0.57±  0.11 | 0.42±  0.13 | 0.31±  0.04 | 0.32±  0.17 | 0.45±  0.22 | 0.54±  0.11 | 0.47±  0.08 | 0.62±  0.16 |
| t18:0 h26:1 hCer | 0.15±  0.02 | 0.03±  0 | 0.04±  0.01 | 0.04±  0.01 | 0.09±  0.03 | 0.13±  0 | 0.14±  0.02 | 0.17±  0.07 |
| t18:1 h16:0 hCer | 0.67±  0.06 | 0.35±  0.07 | 0.38±  0.06 | 0.56±  0.13 | 0.53±  0.03 | 1.14±  0.09 | 1.1±  0.21 | 1.74±  0.58 |
| t18:1 h20:0 hCer | 0.12±  0 | 0.05±  0.01 | 0.04±  0.01 | 0.07±  0.01 | 0.08±  0.01 | 0.1±  0.01 | 0.05±  0.01 | 0.09±  0.06 |
| t18:1 h22:0 hCer | 0.19±  0.02 | 0.09±  0.02 | 0.06±  0.03 | 0.18±  0.04 | 0.16±  0.01 | 0.06±  0.03 | 0.05±  0.02 | 0.05±  0.04 |
| t18:1 h24:0 hCer | 17.14±  1.7 | 7.79±  2.46 | 7.71±  1.68 | 11.2±  2.16 | 9.97±  0.5 | 3.76±  0.47 | 3.71±  0.73 | 4.51±  1.57 |
| t18:1 h24:1 hCer | 11.54±  0.15 | 5.76±  1.58 | 5.43±  1.42 | 9.57±  2.23 | 4.11±  0.37 | 1.84±  0.07 | 1.65±  0.53 | 2.98±  1.06 |
| t18:1 h26:0 hCer | 0.4±  0.06 | 0.28±  0.05 | 0.24±  0.05 | 0.7±  0.35 | 0.63±  0.03 | 0.2±  0.07 | 0.12±  0.03 | 0.16±  0.05 |
| t18:1 h26:1 hCer | 1.32±  0.66 | 1.01±  0.31 | 1.12±  0.38 | 1.6±  0.43 | 0.81±  0.08 | 0.83±  0.08 | 0.71±  0.13 | 1.08±  0.43 |
| d18:0 h16:0 hCer | 0.86±  0.03 | 0.56±  0.09 | 0.46±  0.05 | 0.94±  0.17 | 0.76±  0.16 | 1±  0.05 | 1.1±  0.1 | 1.39±  0.38 |
| d18:0 h24:0 hCer | 0.07±  0 | 0.05±  0 | 0.05±  0 | 0.07±  0.01 | 0.06±  0 | 0.04±  0 | 0.04±  0 | 0.05±  0.01 |
| d18:0 h24:1 hCer | 0.07±  0 | 0.06±  0.01 | 0.06±  0 | 0.09±  0.01 | 0.04±  0 | 0.01±  0 | 0.03±  0 | 0.03±  0.01 |
| d18:1 h16:0 hCer | 0.13±  0.03 | 0.07±  0.03 | 0.09±  0.01 | 0.1±  0.03 | 0.07±  0.02 | 0.1±  0.01 | 0.11±  0.01 | 0.12±  0.03 |
| d18:1 h22:0 hCer | 0.02±  0.02 | 0±  0 | 0±  0 | 0.03±  0.03 | 0.04±  0.04 | 0.04±  0 | 0.02±  0.01 | 0±  0 |
| d18:1 h22:1 hCer | 0.15±  0.02 | 0.1±  0.02 | 0.07±  0.02 | 0.11±  0.02 | 0.11±  0 | 0.1±  0.01 | 0.09±  0.01 | 0.09±  0.02 |
| d18:1 h24:0 hCer | 0±  0 | 0.02±  0.01 | 0.02±  0 | 0.07±  0.06 | 0.05±  0 | 0.02±  0 | 0.02±  0 | 0.01±  0 |
| d18:1 h24:1 hCer | 0.03±  0 | 0.01±  0 | 0.02±  0 | 0.03±  0.01 | 0.07±  0.04 | 0.01±  0 | 0.01±  0 | 0.02±  0 |
| d18:1 h26:0 hCer | 0.01±  0 | 0.09±  0.03 | 0.11±  0.04 | 0.11±  0.09 | 0.14±  0 | 0.15±  0.01 | 0.21±  0.02 | 0.18±  0 |
| d18:1 h26:1 hCer | 0.13±  0.01 | 0.11±  0.03 | 0.04±  0.01 | 0.08±  0.01 | 0.08±  0.02 | 0.1±  0.02 | 0.06±  0.02 | 0.05±  0.01 |
| t18:0 h22:0 GlcCer | 0.26±  0.01 | 0.22±  0.02 | 0.22±  0 | 0.34±  0.06 | 0.28±  0.02 | 0.15±  0 | 0.16±  0 | 0.2±  0.01 |
| t18:0 h24:0 GlcCer | 0.33±  0.04 | 0.25±  0.04 | 0.18±  0.01 | 0.36±  0.06 | 0.34±  0.03 | 0.11±  0 | 0.09±  0.01 | 0.1±  0 |
| t18:0 h24:1 GlcCer | 1.03±  0.05 | 0.81±  0.12 | 0.85±  0.05 | 1.23±  0.24 | 0.89±  0.05 | 0.37±  0.01 | 0.42±  0.04 | 0.46±  0.04 |
| t18:0 h26:0 GlcCer | 0.28±  0.01 | 0.23±  0.02 | 0.23±  0.01 | 0.34±  0.04 | 0.23±  0.03 | 0.14±  0.01 | 0.15±  0 | 0.15±  0.02 |
| t18:0 h26:1 GlcCer | 0.2±  0.01 | 0.13±  0.01 | 0.13±  0.01 | 0.22±  0.03 | 0.11±  0.02 | 0.11±  0.01 | 0.1±  0.02 | 0.12±  0.01 |
| t18:1 h16:0 GlcCer | 15.94±  0.46 | 13.41±  2.15 | 14.87±  0.88 | 20.78±  3.41 | 14.7±  1 | 30.56±  1.52 | 30.75±  1.16 | 39.96±  3.6 |
| t18:1 h18:0 GlcCer | 0.13±  0.01 | 0.12±  0.02 | 0.11±  0 | 0.18±  0.03 | 0.19±  0.01 | 0.09±  0 | 0.08±  0 | 0.07±  0.03 |
| t18:1 h20:0 GlcCer | 0.63±  0.02 | 0.49±  0.07 | 0.44±  0.02 | 0.69±  0.08 | 0.72±  0.06 | 0.48±  0.02 | 0.47±  0.02 | 0.64±  0.05 |
| t18:1 h22:0 GlcCer | 11.59±  0.48 | 9.5±  1.06 | 9.61±  0.51 | 14.71±  2.04 | 11.23±  0.85 | 6.43±  0.16 | 6.27±  0.46 | 9.13±  1.47 |
| t18:1 h22:1 GlcCer | 0.13±  0 | 0.1±  0.01 | 0.11±  0.02 | 0.15±  0.01 | 0.13±  0 | 0.02±  0 | 0.02±  0 | 0.04±  0 |
| t18:1 h24:0 GlcCer | 21.31±  0.69 | 15.76±  1.88 | 12.89±  0.89 | 23.1±  6.45 | 21.12±  0.88 | 6.09±  0.26 | 5.18±  0.35 | 5.97±  0.49 |
| t18:1 h24:1 GlcCer | 34.52±  0.85 | 28.05±  4.21 | 28.2±  0.95 | 42.99±  8.36 | 31.08±  1.94 | 13.15±  0.24 | 13.56±  0.73 | 17.07±  1.69 |
| t18:1 h26:0 GlcCer | 7.6±  0.21 | 5.86±  1.06 | 6.39±  0.46 | 8.09±  1.18 | 5.89±  0.35 | 3.66±  0.24 | 3.98±  0.19 | 4.67±  0.44 |
| t18:1 h26:1 GlcCer | 6.09±  0.15 | 3.66±  0.69 | 3.77±  0.56 | 6.01±  1.08 | 3.65±  0.3 | 2.4±  0.12 | 2.58±  0.26 | 3.59±  0.78 |
| d18:1 h16:0 GlcCer | 28.29±  0.87 | 13.34±  6.94 | 24.74±  2.1 | 25.2±  12.85 | 21.11±  0.99 | 25.7±  0.94 | 24.78±  1.18 | 20.67±  10.78 |
| d18:1 h22:0 GlcCer | 0.52±  0.04 | 0.35±  0.03 | 0.28±  0.04 | 0.56±  0.11 | 0.44±  0.04 | 0.07±  0 | 0.07±  0.01 | 0.09±  0.01 |
| d18:1 h24:0 GlcCer | 2.81±  0.11 | 2.39±  0.39 | 2.71±  0.28 | 3.38±  0.44 | 2.36±  0.18 | 0.31±  0.02 | 0.24±  0.05 | 0.3±  0.05 |
| d18:1 h24:1 GlcCer | 2.33±  0.02 | 1.52±  0.24 | 1.23±  0.17 | 2.31±  0.64 | 1.72±  0.08 | 0.2±  0.02 | 0.13±  0.02 | 0.14±  0.01 |
| d18:1 h26:0 GlcCer | 0.12±  0 | 0.11±  0.01 | 0.11±  0 | 0.24±  0.11 | 0.18±  0.01 | 0.02±  0.01 | 0.01±  0 | 0±  0 |
| d18:1 h26:1 GlcCer | 0.78±  0 | 0.68±  0.12 | 0.72±  0.11 | 0.68±  0.07 | 0.5±  0.13 | 0.36±  0.02 | 0.4±  0.05 | 0.36±  0.03 |

**Supplemental Table 2.** Sphingolipid profiles of 6-week-old WT, *pad4-1*, *eds1-2*, *sid2-1*, *loh1-2*, *loh1-2 pad4-1*, *loh1-2 eds1-2* and *loh1-2 sid2-1* leaves.

| **Species** | **WT** | ***pad4-1*** | ***eds1-2*** | ***sid2-1*** | ***loh1-2*** | ***loh1-2 pad4-1*** | ***loh1-2 eds1-2*** | ***loh1-2 sid2-1*** |
| --- | --- | --- | --- | --- | --- | --- | --- | --- |
| t18:0 | 2.19±  0.3 | 0.36±  0.04 | 0.47±  0.04 | 0.57±  0.03 | 9.93±  0.71 | 2.66±  0.79 | 2.8±  0.13 | 14.32±  1.66 |
| t18:1 | 2.25±  0.01 | 1.48±  0.15 | 1.39±  0.14 | 1.69±  0.15 | 4.67±  0.3 | 3.32±  0.34 | 3.6±  0.17 | 4.63±  0.53 |
| d18:1 | 0.02±  0 | 0.01±  0 | 0.01±  0 | 0.02±  0 | 0.27±  0.01 | 0.03±  0 | 0.04±  0 | 0.15±  0 |
| d18:0 | 0.15±  0.01 | 0.08±  0 | 0.08±  0 | 0.11±  0 | 1.16±  0.11 | 0.15±  0.02 | 0.18±  0 | 0.83±  0.04 |
| t18:0 c16:0 Cer | 2.04±  0.53 | 0.45±  0.08 | 0.42±  0.01 | 0.51±  0.06 | 74.78±  7.3 | 2.28±  0.51 | 2.38±  0.26 | 17.97±  2.53 |
| t18:0 c18:0 Cer | 0.03±  0.01 | 0.05±  0.04 | 0.06±  0.02 | 0.02±  0 | 0.38±  0.03 | 0.01±  0 | 0.05±  0.04 | 0.11±  0.02 |
| t18:0 c20:0 Cer | 0.07±  0 | 0.01±  0 | 0.02±  0 | 0.03±  0 | 0.74±  0.1 | 0.04±  0 | 0.03±  0 | 0.18±  0 |
| t18:0 c20:1 Cer | 0.06±  0 | 0.05±  0 | 0.06±  0 | 0.07±  0 | 0.06±  0 | 0.05±  0 | 0.05±  0 | 0.09±  0 |
| t18:0 c22:0 Cer | 4.18±  0.64 | 1.15±  0.11 | 1.3±  0.2 | 1.49±  0.16 | 7.79±  4.44 | 0.98±  0.48 | 1.19±  0.59 | 4.31±  0.05 |
| t18:0 c24:0 Cer | 0.74±  0.16 | 0.3±  0.02 | 0.34±  0.05 | 0.35±  0.06 | 12.56±  0.75 | 0.3±  0.03 | 0.46±  0.1 | 0.89±  0.1 |
| t18:0 c24:1 Cer | 7.07±  0.75 | 4.02±  0.25 | 4.93±  0.61 | 4.51±  0.64 | 12.58±  0.3 | 3.19±  0.37 | 3.7±  0.17 | 6.28±  0.48 |
| t18:0 c26:0 Cer | 4.46±  0.49 | 2.61±  0.23 | 2.67±  0.49 | 2.79±  0.52 | 16.08±  1.35 | 3.24±  0.28 | 4.45±  0.49 | 7.45±  0.39 |
| t18:0 c26:1 Cer | 0.06±  0 | 0.07±  0.02 | 0.09±  0 | 0.05±  0 | 1.9±  0.19 | 0.12±  0.03 | 0.3±  0.09 | 0.28±  0.05 |
| t18:1 c16:0 Cer | 4.41±  0.82 | 1.65±  0.08 | 1.68±  0.04 | 2.09±  0.13 | 41.23±  0.44 | 6.3±  1.17 | 6.7±  0.72 | 17.05±  1.71 |
| t18:1 c18:0 Cer | 0.03±  0 | 0.02±  0 | 0.02±  0 | 0.01±  0 | 0.17±  0 | 0.03±  0 | 0.02±  0 | 0.06±  0 |
| t18:1 c20:0 Cer | 0.26±  0.02 | 0.13±  0 | 0.11±  0.01 | 0.18±  0.01 | 0.74±  0.04 | 0.16±  0 | 0.17±  0 | 0.33±  0 |
| t18:1 c22:0 Cer | 0.89±  0.06 | 0.52±  0.05 | 0.52±  0 | 0.58±  0.05 | 2.26±  0.07 | 0.57±  0.06 | 0.68±  0.04 | 0.85±  0 |
| t18:1 c24:0 Cer | 13.92±  1.33 | 10.87±  0.39 | 11.89±  1.2 | 12.51±  2.09 | 16.66±  0.28 | 8.12±  0.64 | 8.55±  0.55 | 9.25±  0.48 |
| t18:1 c24:1 Cer | 7.03±  0.46 | 5.94±  0.46 | 6.78±  0.69 | 6.49±  1.05 | 4.64±  0.19 | 3±  0.12 | 2.96±  0.08 | 3.94±  0.04 |
| t18:1 c26:0 Cer | 9.27±  0.93 | 8.17±  0.2 | 9.05±  0.74 | 8.75±  1.48 | 17.64±  0.13 | 8.93±  0.71 | 10.93±  0.82 | 12.39±  0.96 |
| t18:1 c26:1 Cer | 0.42±  0.02 | 0.48±  0.04 | 0.53±  0.06 | 0.48±  0.07 | 0.96±  0.06 | 0.51±  0.03 | 0.68±  0.04 | 0.77±  0.1 |
| d18:0 c16:0 Cer | 0.7±  0.14 | 0.19±  0.02 | 0.18±  0.01 | 0.28±  0 | 6.53±  0.48 | 0.32±  0.06 | 0.33±  0.02 | 2.67±  0.36 |
| d18:0 c18:0 Cer | 0±  0 | 0.02±  0 | 0.02±  0 | 0.04±  0 | 0.04±  0 | 0.01±  0 | 0.02±  0 | 0.02±  0 |
| d18:0 c20:0 Cer | 0.01±  0 | 0.01±  0 | 0.01±  0 | 0.01±  0 | 0.03±  0 | 0.01±  0 | 0.01±  0 | 0.02±  0 |
| d18:1 c16:0 Cer | 0.27±  0.02 | 0.17±  0.01 | 0.18±  0.01 | 0.23±  0.01 | 0.94±  0.02 | 0.23±  0 | 0.26±  0 | 0.57±  0.01 |
| d18:1 c18:0 Cer | 0.06±  0 | 0.06±  0.01 | 0.06±  0 | 0.08±  0 | 0.08±  0 | 0.07±  0 | 0.08±  0 | 0.08±  0 |
| d18:1 c20:0 Cer | 0.01±  0 | 0.02±  0 | 0.01±  0 | 0.03±  0 | 0.05±  0 | 0.02±  0 | 0.04±  0 | 0.03±  0 |
| d18:1 c22:0 Cer | 0.15±  0.01 | 0.14±  0.01 | 0.2±  0.03 | 0.19±  0.01 | 0.15±  0.01 | 0.17±  0 | 0.16±  0 | 0.17±  0.01 |
| d18:1 c24:0 Cer | 0.54±  0.03 | 0.5±  0.01 | 0.49±  0.01 | 0.6±  0.06 | 0.5±  0.01 | 0.5±  0 | 0.56±  0.03 | 0.58±  0.04 |
| d18:1 c24:1 Cer | 0.05±  0.02 | 0.05±  0.02 | 0.06±  0.02 | 0.05±  0.04 | 0.01±  0 | 0.02±  0 | 0.01±  0 | 0.01±  0 |
| d18:1 c26:0 Cer | 0.42±  0.02 | 0.37±  0.02 | 0.35±  0.01 | 0.45±  0.04 | 0.41±  0 | 0.39±  0.01 | 0.45±  0.01 | 0.47±  0.02 |
| d18:1 c26:1 Cer | 0.12±  0 | 0.08±  0.03 | 0.11±  0 | 0.08±  0.03 | 0.01±  0 | 0.12±  0.01 | 0.11±  0 | 0.12±  0.01 |
| t18:0 h16:0 hCer | 0.97±  0.18 | 0.5±  0.1 | 0.5±  0.03 | 0.7±  0.1 | 47.12±  4.28 | 1.6±  0.19 | 2.05±  0.27 | 14.73±  2.63 |
| t18:0 h20:0 hCer | 0.14±  0.02 | 0.08±  0 | 0.05±  0 | 0.12±  0.01 | 1.15±  0.24 | 0.14±  0 | 0.12±  0.01 | 0.99±  0.52 |
| t18:0 h22:0 hCer | 0.88±  0.12 | 0.29±  0.06 | 0.28±  0.02 | 0.59±  0.12 | 15.06±  1.83 | 0.69±  0.05 | 0.71±  0.05 | 3.69±  0.57 |
| t18:0 h24:0 hCer | 9.49±  1.57 | 3.97±  0.36 | 4±  0.39 | 6.78±  1.44 | 48.83±  4.02 | 5.28±  0.3 | 5.97±  0.29 | 49.82±  33.07 |
| t18:0 h24:1 hCer | 4.62±  0.49 | 2.9±  0.15 | 3.5±  0.28 | 3.83±  0.64 | 21.66±  1.32 | 2.57±  0.27 | 2.6±  0.02 | 9.86±  0.64 |
| t18:0 h26:0 hCer | 1.83±  0.21 | 0.74±  0.07 | 0.8±  0.08 | 1.34±  0.25 | 18.08±  1.85 | 1.32±  0.05 | 1.37±  0.08 | 14.84±  8.31 |
| t18:0 h26:1 hCer | 0.19±  0.01 | 0.13±  0.01 | 0.17±  0.02 | 0.23±  0.04 | 2.26±  0.33 | 0.23±  0.03 | 0.28±  0.02 | 0.85±  0.09 |
| t18:1 h16:0 hCer | 2.66±  0.26 | 2.03±  0.11 | 1.96±  0.1 | 2.76±  0.34 | 66.47±  5.95 | 7.55±  0.57 | 8.62±  0.6 | 27.05±  3.3 |
| t18:1 h18:0 hCer | 0.05±  0 | 0.03±  0 | 0.03±  0 | 0.06±  0 | 0.39±  0.05 | 0.03±  0 | 0.04±  0 | 0.13±  0.01 |
| t18:1 h20:0 hCer | 0.37±  0.02 | 0.27±  0.01 | 0.27±  0.02 | 0.4±  0.04 | 3.45±  0.27 | 0.44±  0.05 | 0.41±  0.02 | 1.19±  0.1 |
| t18:1 h22:0 hCer | 0.39±  0.02 | 0.35±  0.01 | 0.37±  0.03 | 0.56±  0.09 | 3.7±  0.46 | 0.49±  0.03 | 0.44±  0.01 | 0.9±  0.09 |
| t18:1 h24:0 hCer | 29.73±  1.7 | 22.63±  1.46 | 22.99±  1.95 | 31.65±  3.58 | 57.26±  1.89 | 17.53±  1.53 | 15.41±  0.45 | 28.96±  0.89 |
| t18:1 h24:1 hCer | 10.82±  0.21 | 11.71±  0.88 | 11.31±  0.54 | 14.17±  1.52 | 23.89±  1.11 | 6.28±  0.15 | 5.66±  0.12 | 10.89±  0.27 |
| t18:1 h26:0 hCer | 0.47±  0.05 | 0.47±  0.06 | 0.48±  0.06 | 0.54±  0.1 | 13.27±  0.91 | 0.65±  0.13 | 1.16±  0.31 | 2.07±  0.34 |
| t18:1 h26:1 hCer | 4.2±  0.09 | 3.7±  0.08 | 3.96±  0.45 | 4.43±  0.51 | 16.61±  1.12 | 3.43±  0.06 | 3.49±  0.05 | 9.09±  0.36 |
| d18:0 h16:0 hCer | 1.22±  0.12 | 0.71±  0.05 | 0.91±  0.01 | 0.94±  0.13 | 8.25±  0.94 | 1.28±  0.03 | 1.59±  0.03 | 4.85±  0.45 |
| d18:0 h20:0 hCer | 0.21±  0.09 | 0.16±  0.04 | 0.09±  0.04 | 0.18±  0.02 | 0.08±  0.03 | 0.09±  0.02 | 0.13±  0.01 | 0.17±  0.06 |
| d18:0 h22:0 hCer | 0.05±  0 | 0.03±  0 | 0.03±  0 | 0.03±  0 | 0.11±  0 | 0.02±  0 | 0.02±  0 | 0.07±  0 |
| d18:0 h24:0 hCer | 0.11±  0 | 0.08±  0 | 0.09±  0 | 0.11±  0 | 0.17±  0 | 0.06±  0 | 0.06±  0 | 0.16±  0.08 |
| d18:0 h24:1 hCer | 0.07±  0 | 0.07±  0 | 0.08±  0 | 0.08±  0.01 | 0.07±  0.01 | 0.04±  0 | 0.02±  0 | 0.04±  0 |
| d18:1 h16:0 hCer | 0.12±  0 | 0.1±  0.07 | 0.14±  0.07 | 0.05±  0.03 | 0.65±  0.14 | 0.23±  0.02 | 0.19±  0.05 | 0.18±  0.01 |
| d18:1 h22:0 hCer | 0.11±  0.01 | 0.09±  0 | 0.09±  0 | 0.12±  0.02 | 0.04±  0.01 | 0.08±  0.02 | 0.09±  0.02 | 0.14±  0.05 |
| d18:1 h22:1 hCer | 0.17±  0.01 | 0.15±  0.01 | 0.14±  0 | 0.13±  0 | 0.31±  0.01 | 0.18±  0.03 | 0.21±  0.01 | 0.21±  0.01 |
| d18:1 h24:1 hCer | 0.15±  0 | 0.14±  0.02 | 0.16±  0 | 0.17±  0.01 | 0.1±  0.01 | 0.04±  0 | 0.02±  0 | 0.06±  0.01 |
| d18:1 h26:0 hCer | 0.48±  0.02 | 0.39±  0.06 | 0.36±  0.02 | 0.49±  0.06 | 0.34±  0.02 | 0.37±  0 | 0.41±  0.02 | 0.38±  0.05 |
| t18:0 h16:0 GlcCer | 0.22±  0 | 0.2±  0.02 | 0.22±  0.01 | 0.23±  0.01 | 0.68±  0.02 | 0.38±  0.04 | 0.44±  0.02 | 0.5±  0.03 |
| t18:0 h18:0 GlcCer | 0.01±  0 | 0±  0 | 0±  0 | 0.01±  0 | 0.02±  0 | 0.02±  0 | 0.02±  0 | 0.05±  0 |
| t18:0 h20:0 GlcCer | 0.01±  0 | 0.02±  0 | 0.01±  0 | 0.01±  0 | 0.02±  0 | 0.01±  0 | 0.01±  0 | 0.01±  0 |
| t18:0 h22:0 GlcCer | 0.2±  0 | 0.19±  0.01 | 0.19±  0 | 0.21±  0.01 | 0.15±  0.01 | 0.14±  0.01 | 0.13±  0 | 0.15±  0 |
| t18:0 h24:0 GlcCer | 0.13±  0 | 0.15±  0.01 | 0.13±  0.01 | 0.15±  0 | 0.14±  0 | 0.08±  0.01 | 0.08±  0 | 0.15±  0.05 |
| t18:0 h24:1 GlcCer | 0.54±  0.02 | 0.6±  0.03 | 0.61±  0.01 | 0.67±  0.01 | 0.5±  0.01 | 0.34±  0.02 | 0.31±  0 | 0.4±  0 |
| t18:0 h26:0 GlcCer | 0.25±  0.02 | 0.24±  0.01 | 0.23±  0 | 0.3±  0.01 | 0.23±  0 | 0.18±  0 | 0.16±  0 | 0.19±  0 |
| t18:0 h26:1 GlcCer | 0.05±  0 | 0.06±  0 | 0.06±  0 | 0.07±  0 | 0.07±  0 | 0.04±  0.01 | 0.05±  0 | 0.06±  0.01 |
| t18:1 h16:0 GlcCer | 12.93±  0.36 | 12.5±  0.49 | 12.75±  0.18 | 14.98±  0.17 | 36.82±  1.49 | 23.52±  1.47 | 27.58±  0.12 | 35.75±  0.22 |
| t18:1 h18:0 GlcCer | 0.13±  0 | 0.13±  0.01 | 0.12±  0.01 | 0.15±  0 | 0.12±  0 | 0.09±  0 | 0.08±  0.01 | 0.1±  0 |
| t18:1 h20:0 GlcCer | 0.47±  0.01 | 0.52±  0.07 | 0.48±  0.02 | 0.54±  0.01 | 0.77±  0.04 | 0.42±  0.01 | 0.43±  0 | 0.59±  0.02 |
| t18:1 h22:0 GlcCer | 9±  0.21 | 8.59±  0.45 | 8.81±  0.2 | 9.43±  0.27 | 6.76±  0.78 | 5.98±  0.34 | 5.58±  0.12 | 6.78±  0.25 |
| t18:1 h22:1 GlcCer | 0.13±  0 | 0.14±  0.01 | 0.13±  0 | 0.17±  0 | 0.07±  0 | 0.05±  0.01 | 0.03±  0 | 0.03±  0 |
| t18:1 h24:0 GlcCer | 9.03±  0.31 | 9.48±  0.38 | 9.71±  0.17 | 11.46±  0.08 | 8.73±  0.21 | 5.58±  0.63 | 4.19±  0.17 | 5.16±  0.1 |
| t18:1 h24:1 GlcCer | 19.62±  0.4 | 20.7±  0.95 | 20.16±  0.41 | 22.55±  0.25 | 16.57±  0.47 | 11.99±  0.99 | 10.2±  0.08 | 13±  0.08 |
| t18:1 h26:0 GlcCer | 7.16±  0.25 | 6.94±  0.33 | 6.95±  0.19 | 7.99±  0.25 | 6.23±  0.19 | 4.67±  0.26 | 4.73±  0.12 | 5.65±  0.15 |
| t18:1 h26:1 GlcCer | 2.01±  0.04 | 2.23±  0.24 | 2.26±  0.11 | 2.41±  0.11 | 2.91±  0.13 | 1.63±  0.1 | 1.9±  0.06 | 2.42±  0.1 |
| d18:0 h16:0 GlcCer | 1.79±  0.08 | 1.53±  0.15 | 1.74±  0.09 | 1.73±  0.08 | 1.6±  0.12 | 1.45±  0.09 | 1.45±  0.08 | 1.55±  0.11 |
| d18:0 h26:0 GlcCer | 0.02±  0 | 0.03±  0 | 0.03±  0 | 0.03±  0 | 0.05±  0 | 0.03±  0 | 0.04±  0 | 0.03±  0 |
| d18:0 h26:1 GlcCer | 0.08±  0 | 0.08±  0 | 0.08±  0 | 0.1±  0.01 | 0.09±  0.02 | 0.07±  0 | 0.07±  0 | 0.1±  0.01 |
| d18:1 h16:0 GlcCer | 26.33±  0.75 | 25.16±  0.72 | 26.65±  0.86 | 29.77±  0.58 | 27.31±  0.45 | 24.6±  0.35 | 25.74±  0.45 | 31.19±  0.25 |
| d18:1 h22:0 GlcCer | 0.5±  0.02 | 0.5±  0 | 0.53±  0.05 | 0.51±  0.01 | 0.18±  0.01 | 0.18±  0.04 | 0.1±  0 | 0.14±  0.01 |
| d18:1 h24:0 GlcCer | 2.99±  0.19 | 2.43±  0.08 | 2.73±  0.18 | 3.17±  0.06 | 0.56±  0.08 | 0.64±  0.25 | 0.25±  0.02 | 0.47±  0.11 |
| d18:1 h24:1 GlcCer | 0.85±  0.02 | 0.89±  0.02 | 0.91±  0.03 | 1.04±  0.02 | 0.39±  0.01 | 0.28±  0.07 | 0.12±  0 | 0.14±  0 |
| d18:1 h26:0 GlcCer | 0.08±  0.01 | 0.09±  0.02 | 0.1±  0.01 | 0.09±  0.01 | 0.16±  0 | 0.04±  0.01 | 0.05±  0.01 | 0.03±  0.02 |
| d18:1 h26:1 GlcCer | 0.47±  0.02 | 0.46±  0.04 | 0.5±  0.01 | 0.5±  0.02 | 0.14±  0.02 | 0.14±  0.03 | 0.1±  0.01 | 0.1±  0.01 |
| d18:0 h16:0 GIPC | 0.38±  0.03 | 0.62±  0.2 | 0.46±  0.16 | 1.81±  0.99 | 6.66±  0.9 | 2.98±  0.58 | 3.38±  0.51 | 3.98±  0.73 |
| d18:0 h20:0 GIPC | 0.19±  0.12 | 0.38±  0.27 | 1.29±  0.6 | 0.43±  0.33 | 0.38±  0.2 | 0.1±  0.03 | 0.16±  0.03 | 0.29±  0.12 |
| d18:0 h22:0 GIPC | 2.69±  0.32 | 2.17±  0.01 | 1.69±  0.22 | 0.64±  0.12 | 1.44±  0.05 | 1.27±  0.06 | 1.34±  0.16 | 0.4±  0.08 |
| d18:0 h26:1 GIPC | 0.34±  0.21 | 0.68±  0.08 | 0.99±  0.08 | 0.77±  0.03 | 0.76±  0.3 | 1.26±  0.22 | 1.18±  0.45 | 0.7±  0.06 |
| d18:0 h24:1 GIPC | 4.66±  3.91 | 3.74±  3.2 | 0.97±  0.09 | 3.72±  2.64 | 1.7±  1.32 | 0.33±  0.11 | 2.52±  1.01 | 1.39±  0.94 |
| d18:0 h24:0 GIPC | 2.9±  1.94 | 0.93±  0.56 | 1.43±  0.53 | 0.59±  0.3 | 0.27±  0.06 | 0.51±  0.03 | 0.44±  0.14 | 0.25±  0.05 |
| d18:0 h26:0 GIPC | 0.21±  0.06 | 0.33±  0.15 | 0.39±  0.04 | 0.35±  0.13 | 0.41±  0.17 | 0.71±  0.33 | 0.21±  0.06 | 0.46±  0.15 |
| d18:1 h16:0 GIPC | 2.82±  0.64 | 3.16±  0.55 | 3.25±  1.01 | 8.03±  5.5 | 11.14±  3.23 | 11.88±  1.16 | 15±  1.73 | 13.43±  2.03 |
| t18:0 h16:0 GIPC | 0.63±  0.25 | 0.41±  0.1 | 3.03±  2.6 | 1.71±  1.35 | 7.92±  0.77 | 1.64±  0.16 | 14.75±  6.76 | 2.47±  0.28 |
| t18:0 h20:0 GIPC | 0.09±  0.04 | 0.11±  0.03 | 0.12±  0.03 | 0.13±  0.01 | 0.1±  0.04 | 0.07±  0 | 0.16±  0.08 | 0.11±  0.01 |
| t18:0 h22:0 GIPC | 93.92±  45.52 | 10.78±  0.38 | 8.45±  4.02 | 3.91±  2.23 | 0.45±  0.05 | 8.54±  3.18 | 4.35±  3.51 | 5.99±  5.38 |
| t18:0 h24:0 GIPC | 4.62±  0.51 | 2.53±  1.01 | 1.66±  0.78 | 1.74±  1.33 | 2.03±  1.13 | 1.45±  0.59 | 1.32±  0.47 | 0.42±  0.21 |
| t18:0 h24:1 GIPC | 29.71±  0.87 | 28.38±  1.92 | 33.27±  4.37 | 34.69±  4.36 | 19.47±  3.36 | 21.22±  0.58 | 17.83±  0.78 | 19.09±  1.06 |
| t18:0 h26:1 GIPC | 1.99±  1.62 | 0.89±  0.6 | 3.96±  3.47 | 2.66±  1.76 | 1.7±  0.15 | 3.19±  2.29 | 1.73±  0.53 | 2.07±  0.09 |
| t18:0 h26:0 GIPC | 0.09±  0.03 | 0.11±  0.07 | 0.45±  0.42 | 0.41±  0.11 | 0.36±  0.15 | 0.3±  0.2 | 0.24±  0.16 | 0.15±  0.02 |
| t18:1 h16:0 GIPC | 1.78±  0.04 | 1.99±  0.14 | 2.63±  0.65 | 1.55±  0.07 | 12.77±  1.14 | 5.65±  0.17 | 7.73±  0.35 | 9.64±  0.37 |
| t18:1 h20:0 GIPC | 0.23±  0.06 | 0.71±  0.59 | 0.17±  0.02 | 0.21±  0.1 | 0.3±  0.11 | 0.12±  0 | 1.42±  0.64 | 0.3±  0.03 |
| t18:1 h22:0 GIPC | 2.97±  1.03 | 3±  0.19 | 3.94±  0.42 | 3±  0.24 | 3.35±  0.39 | 1.92±  0.47 | 3.32±  0.38 | 1.45±  0.6 |
| t18:1 h26:1 GIPC | 0.5±  0.13 | 0.49±  0.14 | 0.35±  0.14 | 0.88±  0.15 | 0.26±  0.14 | 1±  0.54 | 0.83±  0.04 | 0.22±  0.05 |
| t18:1 h24:0 GIPC | 22.21±  0.32 | 20.04±  1.17 | 22.83±  3.2 | 23.27±  2.91 | 13.36±  1.99 | 14.3±  0.74 | 12.22±  0.73 | 12.7±  0.69 |
| t18:1 h24:1 GIPC | 35.55±  0.46 | 43.6±  1.26 | 45±  5.24 | 39.5±  4.22 | 16.79±  4.18 | 28.02±  3.16 | 20.75±  1.02 | 18.58±  0.91 |
| t18:1 h26:0 GIPC | 0.1±  0.02 | 0.18±  0.02 | 2.64±  2.31 | 0.21±  0.04 | 0.9±  0.21 | 2.12±  1.52 | 4.08±  1.77 | 1.31±  1.08 |

**Supplemental Table 3.** Sphingolipid profiles of 3-week-old WT, *pad4-1*, *eds1-2*, and *sid2-1* leaves before FB1 treatment.

| **Species** | **Col-0d** | ***pad4-1*-0d** | ***eds1-2*-0d** | ***sid2-1*-0d** |
| --- | --- | --- | --- | --- |
| d18:0 LCB | 0.06±0.01 | 0.05±0.01 | 0.04±0 | 0.05±0 |
| d18:1 LCB | 0.01±0 | 0±0 | 0±0 | 0.01±0 |
| t18:0 LCB | 0.69±0.22 | 0.48±0.17 | 0.36±0.04 | 0.68±0.09 |
| t18:1 LCB | 1.16±0.11 | 0.83±0.01 | 0.83±0.03 | 0.83±0.04 |
| d18:0 c16:0 Cer | 0.32±0.03 | 0.19±0.03 | 0.18±0.02 | 0.29±0 |
| d18:0 c22:0 Cer | 0.06±0 | 0.06±0.02 | 0.04±0.01 | 0.06±0 |
| d18:0 c24:0 Cer | 0.14±0.03 | 0.09±0.01 | 0.13±0.02 | 0.12±0.02 |
| d18:0 c24:1 Cer | 0.03±0.01 | 0.04±0.01 | 0.12±0.07 | 0.05±0.01 |
| d18:1 c16:0 Cer | 0.19±0.03 | 0.13±0.03 | 0.1±0.02 | 0.14±0.03 |
| d18:1 c20:0 Cer | 0.11±0.02 | 0.06±0 | 0.41±0.24 | 0.15±0.02 |
| d18:1 c24:0 Cer | 0.07±0.01 | 0.11±0.02 | 0.29±0.17 | 0.12±0.02 |
| d18:1 c26:0 Cer | 0.2±0.05 | 0.26±0.08 | 0.21±0 | 0.18±0.03 |
| d18:1 c26:1 Cer | 0.06±0.01 | 0.17±0.02 | 0.1±0.01 | 0.05±0 |
| t18:0 c16:0 Cer | 0.65±0.09 | 0.43±0.08 | 0.38±0.04 | 1.16±0.04 |
| t18:0 c18:0 Cer | 0.06±0.02 | 0.04±0.01 | 0.04±0.02 | 0.08±0.02 |
| t18:0 c22:0 Cer | 1.45±0.03 | 0.98±0.12 | 1.15±0.03 | 1.66±0.21 |
| t18:0 c24:0 Cer | 9.76±0.55 | 10.03±0.79 | 10.23±1.06 | 14.36±1.06 |
| t18:0 c24:1 Cer | 10.42±0.91 | 11.38±0.83 | 11.3±1.47 | 16.07±0.96 |
| t18:0 c26:0 Cer | 5.49±0.06 | 6.85±1.15 | 6.23±1.18 | 9.78±1.12 |
| t18:0 c26:1 Cer | 1.12±0.07 | 1.29±0.18 | 1.33±0.16 | 1.57±0.17 |
| t18:1 c16:0 Cer | 1.38±0.09 | 1.16±0.01 | 0.8±0.06 | 1.46±0.06 |
| t18:1 c20:0 Cer | 0.16±0.02 | 0.05±0.01 | 0.15±0.03 | 0.3±0.04 |
| t18:1 c22:0 Cer | 0.88±0.23 | 0.39±0.03 | 0.95±0.33 | 1.68±0.02 |
| t18:1 c22:1 Cer | 0.09±0.02 | 0.14±0.04 | 0.06±0.02 | 0.09±0.01 |
| t18:1 c24:0 Cer | 4.01±2.34 | 8.11±2.68 | 1.44±0.15 | 1.97±0.23 |
| t18:1 c24:1 Cer | 2.72±0.52 | 1.02±0.15 | 2.74±0.84 | 3.17±0.01 |
| t18:1 c26:0 Cer | 11.88±2.51 | 14.53±0.66 | 16.9±2.49 | 21.95±2.51 |
| t18:1 c26:1 Cer | 1.52±0.77 | 3.41±0.43 | 0.37±0 | 0.7±0.06 |
| d18:0 h16:0 hCer | 0.88±0.05 | 0.52±0.03 | 0.62±0.05 | 1.02±0.05 |
| d18:0 h24:0 hCer | 0.12±0.05 | 0.1±0.02 | 0.32±0.19 | 0.18±0.03 |
| d18:1 h16:0 hCer | 0.61±0.03 | 0.58±0.03 | 0.57±0.13 | 1.04±0.06 |
| d18:1 h22:0 hCer | 0.1±0.02 | 0.17±0.02 | 0.19±0.04 | 0.12±0.02 |
| d18:1 h24:0 hCer | 2.09±0.14 | 2.13±0.26 | 1.69±0.48 | 2.74±0.25 |
| d18:1 h24:1 hCer | 0.22±0.04 | 0.19±0.06 | 0.38±0.03 | 0.34±0.08 |
| t18:0 h16:0 hCer | 0.84±0.07 | 0.65±0.2 | 0.47±0.16 | 1.4±0.17 |
| t18:0 h20:0 hCer | 0.13±0.01 | 0.09±0.01 | 0.1±0.02 | 0.18±0.04 |
| t18:0 h22:0 hCer | 2.02±0.14 | 1.53±0.53 | 1.44±0.31 | 2.95±0.32 |
| t18:0 h24:0 hCer | 1.65±0.75 | 2.4±0.77 | 0.67±0.13 | 1.17±0.14 |
| t18:0 h24:1 hCer | 2.98±1.14 | 0.75±0.25 | 3.23±1.01 | 6.26±0.65 |
| t18:0 h26:0 hCer | 2.56±0.41 | 2.86±0.36 | 2.15±0.26 | 3.3±0.42 |
| t18:1 h16:0 hCer | 5.94±0.25 | 5.92±0.17 | 6.01±0.11 | 10.47±0.41 |
| t18:1 h20:0 hCer | 0.89±0.14 | 0.64±0.11 | 0.83±0.17 | 1.43±0.01 |
| t18:1 h22:0 hCer | 6.01±0.15 | 9.23±1.03 | 4.55±0.57 | 7.17±0.31 |
| t18:1 h24:0 hCer | 38.68±5.5 | 45.3±4.26 | 37.74±3.24 | 48.87±4.99 |
| t18:1 h24:1 hCer | 12.75±2.55 | 8.12±1.6 | 10.05±3.47 | 18.31±0.49 |
| t18:1 h26:0 hCer | 19.38±3.38 | 20.89±1.35 | 20.71±1.55 | 28.35±0.94 |
| t18:1 h26:1 hCer | 5.13±0.46 | 6.04±0.64 | 4.72±0.24 | 6.1±0.55 |
| d18:0 h16:0 GlcCer | 0.46±0.14 | 0.12±0.04 | 0.53±0.19 | 0.71±0.06 |
| d18:1 h16:0 GlcCer | 157.19±8.63 | 104.85±3.77 | 123.63±7.73 | 124.3±8.96 |
| d18:1 h22:0 GlcCer | 0.19±0.03 | 0.1±0.02 | 0.13±0.01 | 0.2±0.01 |
| d18:1 h24:0 GlcCer | 0.38±0.05 | 0.23±0.11 | 0.44±0.04 | 0.32±0.05 |
| d18:1 h24:1 GlcCer | 3.43±0.72 | 1.7±0.37 | 3.13±0.41 | 2.53±0.11 |
| d18:1 h26:1 GlcCer | 0.16±0.02 | 0.12±0.03 | 0.12±0.02 | 0.08±0.01 |
| t18:0 h24:0 GlcCer | 0.24±0.05 | 0.05±0 | 0.18±0.02 | 0.25±0.04 |
| t18:1 h16:0 GlcCer | 28.24±1.2 | 24.69±1 | 24.2±0.88 | 25.78±0.94 |
| t18:1 h18:0 GlcCer | 0.31±0.06 | 0.19±0.03 | 0.24±0.03 | 0.32±0 |
| t18:1 h20:0 GlcCer | 0.49±0.05 | 0.44±0.03 | 0.41±0.02 | 0.41±0 |
| t18:1 h22:0 GlcCer | 10.73±2.88 | 4.82±0.31 | 10.12±2.07 | 13.57±0.54 |
| t18:1 h22:1 GlcCer | 0.4±0.07 | 0.35±0.01 | 0.39±0.06 | 0.39±0.01 |
| t18:1 h24:0 GlcCer | 28.4±5.06 | 15.88±2.61 | 24.6±3.38 | 28.58±1.62 |
| t18:1 h24:1 GlcCer | 38.03±13.92 | 10.96±0.87 | 41.19±12.8 | 54.95±3.87 |
| t18:1 h26:0 GlcCer | 3±0.38 | 4.66±0.26 | 2.68±0.51 | 2.47±0.09 |
| t18:1 h26:1 GlcCer | 4.54±1.07 | 2.26±0.09 | 4.06±0.91 | 4.73±0.36 |
| d18:1 h16:0 GIPC | 21.6±4.3 | 16.14±1.07 | 20.15±1.57 | 24.32±1.25 |
| d18:0 h16:0 GIPC | 5.55±0.56 | 4.08±0.69 | 3.64±0.61 | 5.04±0.97 |
| t18:1 h16:0 GIPC | 33.29±1.47 | 26.23±1.6 | 29.06±1.13 | 40.33±3.28 |
| t18:0 h16:0 GIPC | 5.42±0.3 | 4.1±0.47 | 3.33±0.44 | 6.19±0.42 |
| t18:1 h20:0 GIPC | 2.35±0.5 | 1.95±0.1 | 1.37±0.18 | 2.14±0.29 |
| d18:0 h22:0 GIPC | 3.04±0.57 | 2.1±0.46 | 2.59±0.57 | 2.77±0.41 |
| t18:1 h22:0 GIPC | 32±2.67 | 26.44±2.3 | 21.29±1.39 | 31.45±5.2 |
| t18:0 h22:0 GIPC | 8.72±0.87 | 6.93±0.28 | 5.37±0.23 | 7.92±0.74 |
| d18:0 h24:1 GIPC | 0.91±0.44 | 3.05±2.08 | 0.82±0.04 | 0.97±0.11 |
| d18:0 h24:0 GIPC | 3.86±0.52 | 2.43±0.37 | 2.01±0.61 | 2.69±0.26 |
| t18:1 h24:1 GIPC | 68.23±6.29 | 59.1±3.37 | 54.22±3.75 | 66.03±8.25 |
| t18:1 h24:0 GIPC | 73.19±8.7 | 61.09±6.4 | 50.56±5.85 | 72.14±12.01 |
| t18:0 h24:1 GIPC | 25.75±0.42 | 20.99±1.71 | 21.29±1.37 | 29.06±4.59 |
| t18:0 h24:0 GIPC | 25.34±3.75 | 20.46±1.83 | 16.07±2.08 | 24.28±5.29 |
| d18:0 h26:1 GIPC | 0.77±0.34 | 0.83±0.25 | 1.37±0.04 | 1.72±0.28 |
| d18:0 h26:0 GIPC | 0.93±0.39 | 0.68±0.23 | 0.41±0.07 | 0.61±0.16 |
| t18:1 h26:1 GIPC | 9.51±0.75 | 8.16±1.14 | 7.18±1.08 | 9.76±1.75 |
| t18:1 h26:0 GIPC | 30.3±4.13 | 25.5±3.11 | 17.62±2.51 | 30.07±7.86 |
| t18:0 h26:1 GIPC | 45.47±6.2 | 38.25±4.67 | 26.44±3.78 | 44.94±11.76 |
| t18:0 h26:0 GIPC | 5.87±2.13 | 2.09±0.26 | 2.72±0.8 | 7.7±2.15 |

**Supplemental Table 4.** Sphingolipid profiles of 3-week-old WT, *pad4-1*, *eds1-2*, and *sid2-1* leaves 24 hours after FB1 treatment.

| **Species** | **Col-1d** | ***pad4-1*-24 h** | ***eds1-2*-24 h** | ***sid2-1*-24 h** |
| --- | --- | --- | --- | --- |
| d18:0 LCB | 5.65±0.7 | 6.22±0.87 | 3.49±0.26 | 4.73±0.54 |
| d18:1 LCB | 0.27±0.01 | 0.24±0.01 | 0.21±0.01 | 0.25±0.01 |
| t18:0 LCB | 36.11±1.59 | 34.22±2.43 | 31.19±0.49 | 37.63±4.35 |
| t18:1 LCB | 3.06±0.36 | 3.46±0.43 | 2.64±0.02 | 2.99±0.55 |
| d18:0 c16:0 Cer | 0.38±0.04 | 0.33±0.07 | 0.3±0.01 | 0.5±0.05 |
| d18:0 c22:0 Cer | 0.02±0 | 0.02±0 | 0.04±0.01 | 0.08±0.01 |
| d18:0 c24:0 Cer | 0.1±0.03 | 0.09±0.01 | 0.07±0.02 | 0.17±0.02 |
| d18:0 c24:1 Cer | 0.02±0.01 | 0.02±0 | 0.02±0 | 0.03±0 |
| d18:1 c16:0 Cer | 0.06±0 | 0.07±0.02 | 0.05±0.01 | 0.12±0.05 |
| d18:1 c20:0 Cer | 0.07±0.02 | 0.09±0.01 | 0.1±0.02 | 0.16±0.01 |
| d18:1 c24:0 Cer | 0.1±0.03 | 0.06±0.01 | 0.1±0.01 | 0.07±0.02 |
| d18:1 c26:0 Cer | 0.21±0.01 | 0.12±0.01 | 0.15±0 | 0.19±0.01 |
| d18:1 c26:1 Cer | 0.03±0 | 0.07±0 | 0.04±0.01 | 0.07±0.02 |
| t18:0 c16:0 Cer | 0.73±0.16 | 0.61±0.08 | 0.54±0.01 | 1.33±0.12 |
| t18:0 c18:0 Cer | 0.04±0 | 0.03±0 | 0.04±0.02 | 0.04±0.01 |
| t18:0 c22:0 Cer | 0.76±0.16 | 0.78±0.18 | 0.68±0.06 | 0.92±0.15 |
| t18:0 c24:0 Cer | 9.35±0.28 | 8.18±1.48 | 8.44±1.34 | 11.5±1.1 |
| t18:0 c24:1 Cer | 10.11±0.82 | 9.46±1.71 | 9.01±1.31 | 10.51±1.23 |
| t18:0 c26:0 Cer | 6.59±0.06 | 5.35±0.69 | 5.33±0.85 | 6.64±0.55 |
| t18:0 c26:1 Cer | 1.02±0.17 | 1.1±0.16 | 1.14±0.13 | 1.33±0.21 |
| t18:1 c16:0 Cer | 0.69±0.08 | 0.35±0.03 | 0.38±0.04 | 0.97±0.06 |
| t18:1 c20:0 Cer | 0.03±0.01 | 0.07±0.02 | 0.06±0.01 | 0.07±0.02 |
| t18:1 c22:0 Cer | 0.35±0.13 | 0.59±0.08 | 0.56±0.05 | 0.62±0.12 |
| t18:1 c22:1 Cer | 0.09±0.04 | 0.05±0.01 | 0.06±0.02 | 0.18±0 |
| t18:1 c24:0 Cer | 2.28±0.97 | 0.85±0.16 | 1.28±0.09 | 1.45±0.13 |
| t18:1 c24:1 Cer | 1.03±0.25 | 1.39±0.16 | 1.45±0.08 | 1.62±0.17 |
| t18:1 c26:0 Cer | 13.02±1.07 | 11.42±2.02 | 11.78±1.6 | 14.57±1.21 |
| t18:1 c26:1 Cer | 0.87±0.37 | 0.22±0.04 | 0.55±0.12 | 1.04±0.12 |
| d18:0 h16:0 hCer | 0.67±0.09 | 0.66±0.13 | 0.5±0.04 | 0.83±0.04 |
| d18:0 h24:0 hCer | 0.1±0.02 | 0.11±0.03 | 0.06±0 | 0.13±0.01 |
| d18:1 h16:0 hCer | 0.33±0.05 | 0.35±0.04 | 0.37±0.09 | 0.7±0.05 |
| d18:1 h22:0 hCer | 0.08±0.02 | 0.04±0 | 0.05±0 | 0.07±0.02 |
| d18:1 h24:0 hCer | 1.33±0.14 | 1.48±0.27 | 1.28±0.2 | 1.79±0.17 |
| d18:1 h24:1 hCer | 0.11±0.05 | 0.09±0.02 | 0.06±0.02 | 0.08±0 |
| t18:0 h16:0 hCer | 0.87±0.15 | 0.65±0.06 | 0.67±0.04 | 1.43±0.11 |
| t18:0 h20:0 hCer | 0.1±0.01 | 0.19±0.01 | 0.17±0 | 0.27±0.05 |
| t18:0 h22:0 hCer | 1.03±0.19 | 1.47±0.13 | 1.46±0.06 | 2.57±0.19 |
| t18:0 h24:0 hCer | 0.91±0.06 | 0.84±0.03 | 0.68±0.04 | 1.1±0.13 |
| t18:0 h24:1 hCer | 1.5±1.14 | 3.36±0.2 | 3.78±0.14 | 4.98±0.58 |
| t18:0 h26:0 hCer | 2.26±0.32 | 2.58±0.59 | 2.19±0.26 | 3.69±0.14 |
| t18:1 h16:0 hCer | 4.26±0.14 | 3.01±0.48 | 2.84±0.03 | 5.7±0.56 |
| t18:1 h20:0 hCer | 0.64±0.03 | 0.44±0.06 | 0.37±0.08 | 0.63±0.01 |
| t18:1 h22:0 hCer | 3.66±0.35 | 2.12±0.19 | 2.37±0.19 | 4.42±0.16 |
| t18:1 h24:0 hCer | 24.66±1.88 | 21.58±3.97 | 19.88±0.83 | 26.63±1.82 |
| t18:1 h24:1 hCer | 4.16±1.79 | 4.76±0.94 | 4.69±0.24 | 10.79±1.4 |
| t18:1 h26:0 hCer | 12.52±0.23 | 12.75±2.13 | 10.14±0.28 | 16.35±0.77 |
| t18:1 h26:1 hCer | 3.07±0.29 | 2.53±0.18 | 2.39±0.34 | 3.61±0.51 |
| d18:0 h16:0 GlcCer | 0.3±0.14 | 0.55±0.02 | 0.59±0.05 | 0.71±0.04 |
| d18:1 h16:0 GlcCer | 91.58±6.6 | 93.37±3.15 | 96.9±6.54 | 118.49±4.09 |
| d18:1 h22:0 GlcCer | 0.1±0.04 | 0.14±0.02 | 0.13±0.01 | 0.22±0.03 |
| d18:1 h24:0 GlcCer | 0.27±0 | 0.3±0.03 | 0.31±0.01 | 0.35±0.07 |
| d18:1 h24:1 GlcCer | 1.49±0.49 | 2.7±0.23 | 2.42±0.1 | 2.69±0.18 |
| d18:1 h26:1 GlcCer | 0.05±0.01 | 0.08±0.02 | 0.07±0.01 | 0.09±0.01 |
| t18:0 h24:0 GlcCer | 0.09±0.05 | 0.15±0 | 0.21±0.02 | 0.25±0 |
| t18:1 h16:0 GlcCer | 19.83±0.49 | 17.81±0.43 | 17.6±0.54 | 23.21±1.4 |
| t18:1 h18:0 GlcCer | 0.22±0.01 | 0.21±0.01 | 0.21±0.02 | 0.27±0.01 |
| t18:1 h20:0 GlcCer | 0.37±0.01 | 0.39±0.02 | 0.34±0.02 | 0.47±0.04 |
| t18:1 h22:0 GlcCer | 7.19±1.98 | 11.21±0.51 | 10.69±0.51 | 13.33±0.64 |
| t18:1 h22:1 GlcCer | 0.33±0 | 0.34±0.01 | 0.37±0.03 | 0.45±0.03 |
| t18:1 h24:0 GlcCer | 13.94±3.77 | 21.82±0.3 | 21.29±1.37 | 27.79±0.91 |
| t18:1 h24:1 GlcCer | 22.55±11.47 | 44.44±0.35 | 46.83±2.86 | 58.93±2.73 |
| t18:1 h26:0 GlcCer | 2.84±0.53 | 1.98±0.32 | 1.95±0.25 | 2.69±0.07 |
| t18:1 h26:1 GlcCer | 2.47±0.69 | 4.18±0.39 | 4.05±0.3 | 4.79±0.37 |
| d18:1 h16:0 GIPC | 17.83±1.57 | 14.07±1.09 | 14.24±0.71 | 22.97±3.49 |
| d18:0 h16:0 GIPC | 5.22±0.86 | 4.62±0.31 | 5.25±1.26 | 7.67±0.53 |
| t18:1 h16:0 GIPC | 23.36±1.25 | 19.46±0.83 | 18.4±1.27 | 30.28±2.69 |
| t18:0 h16:0 GIPC | 4.01±0.45 | 3.58±0.37 | 3±0.33 | 6.7±1.9 |
| t18:1 h20:0 GIPC | 1.66±0.41 | 1.23±0.23 | 1.25±0.3 | 1.87±0.25 |
| d18:0 h22:0 GIPC | 1.15±0.32 | 1.14±0.18 | 0.98±0.13 | 2.19±0.19 |
| t18:1 h22:0 GIPC | 21.8±1.33 | 18.4±1.22 | 16.38±0.51 | 23.36±1.48 |
| t18:0 h22:0 GIPC | 5.1±0.44 | 4.45±0.2 | 3.89±0.29 | 6.13±0.17 |
| d18:0 h24:1 GIPC | 0.56±0.16 | 0.27±0.11 | 0.54±0.19 | 0.77±0.16 |
| d18:0 h24:0 GIPC | 1.25±0.4 | 0.98±0.11 | 1.63±0.17 | 1.65±0.35 |
| t18:1 h24:1 GIPC | 49.4±1.73 | 44.1±0.41 | 37.44±0.55 | 49.2±5.13 |
| t18:1 h24:0 GIPC | 53.26±1.5 | 44.81±2.11 | 35.51±0.67 | 57.87±4.75 |
| t18:0 h24:1 GIPC | 14.04±1.77 | 15.41±1.83 | 11.87±1.09 | 18.46±1.67 |
| t18:0 h24:0 GIPC | 17.58±1.34 | 13.94±0.47 | 11.19±0.52 | 23.81±2.99 |
| d18:0 h26:1 GIPC | 0.7±0.11 | 0.72±0.15 | 0.36±0.12 | 0.68±0.27 |
| d18:0 h26:0 GIPC | 1.08±0.33 | 0.27±0.13 | 0.31±0.04 | 0.55±0.17 |
| t18:1 h26:1 GIPC | 7.8±0.27 | 5.97±0.46 | 4.73±0.38 | 7.91±0.63 |
| t18:1 h26:0 GIPC | 22.31±0.76 | 15.18±2.06 | 13.2±1.16 | 25.18±2.84 |
| t18:0 h26:1 GIPC | 33.08±0.78 | 23.28±2.6 | 19.81±1.75 | 37.72±4.29 |
| t18:0 h26:0 GIPC | 7.01±0.29 | 3.86±1.46 | 1.96±0.8 | 4.18±1.39 |

**Supplemental Table 5.** Sphingolipid profiles of 3-week-old WT, *pad4-1*, *eds1-2*, and *sid2-1* leaves 72 hours after FB1 treatment.

| **Species** | **Col-72 h** | ***pad4-1*-72 h** | ***eds1-2*-72 h** | ***sid2-1*-72 h** |
| --- | --- | --- | --- | --- |
| d18:0 LCB | 8.67±1.37 | 9.1±0.5 | 9.17±0.18 | 12.27±1.27 |
| d18:1 LCB | 0.39±0 | 0.23±0.01 | 0.29±0.01 | 0.42±0.01 |
| t18:0 LCB | 42.63±4.46 | 41.51±0.47 | 42.96±1.31 | 50.63±1.01 |
| t18:1 LCB | 6.79±0.89 | 6.56±0.46 | 6.06±0.24 | 6.46±0.38 |
| d18:0 c16:0 Cer | 2.77±0.5 | 1.26±0.21 | 1.07±0.16 | 1.36±0.27 |
| d18:0 c22:0 Cer | 0.06±0 | 0.03±0 | 0.06±0.01 | 0.09±0 |
| d18:0 c24:0 Cer | 0.12±0.01 | 0.12±0.03 | 0.14±0.02 | 0.16±0.02 |
| d18:0 c24:1 Cer | 0.08±0.02 | 0.07±0.02 | 0.08±0 | 0.08±0.01 |
| d18:1 c16:0 Cer | 0.3±0.06 | 0.2±0.05 | 0.26±0.04 | 0.28±0.06 |
| d18:1 c20:0 Cer | 0.06±0.01 | 0.08±0.02 | 0.47±0.34 | 0.12±0.02 |
| d18:1 c24:0 Cer | 0.14±0 | 0.07±0.01 | 0.12±0.03 | 0.11±0.01 |
| d18:1 c26:0 Cer | 0.13±0.02 | 0.15±0 | 0.17±0.02 | 0.21±0.02 |
| d18:1 c26:1 Cer | 0.07±0 | 0.08±0 | 0.25±0.18 | 0.07±0.01 |
| t18:0 c16:0 Cer | 6.34±1.31 | 2.79±0.44 | 2.67±0.26 | 3.15±0.68 |
| t18:0 c18:0 Cer | 0.03±0 | 0.02±0 | 0.07±0.05 | 0.03±0 |
| t18:0 c22:0 Cer | 1.19±0.27 | 1.02±0.12 | 1.33±0.1 | 1.66±0.28 |
| t18:0 c24:0 Cer | 13.85±1.91 | 12.27±1.49 | 15.36±0.13 | 15.7±1.26 |
| t18:0 c24:1 Cer | 19.58±3.4 | 14.93±0.99 | 18.56±1.3 | 17.65±2.48 |
| t18:0 c26:0 Cer | 8.73±0.95 | 8.55±0.83 | 10.12±1 | 10.07±0.82 |
| t18:0 c26:1 Cer | 2.38±0.38 | 1.66±0.22 | 2.17±0.19 | 2.23±0.28 |
| t18:1 c16:0 Cer | 2.02±0.34 | 1.41±0.31 | 1.44±0.24 | 1.47±0.16 |
| t18:1 c20:0 Cer | 0.09±0.02 | 0.11±0 | 0.1±0.03 | 0.19±0.05 |
| t18:1 c22:0 Cer | 0.55±0.16 | 0.84±0.08 | 0.66±0.13 | 1.04±0.21 |
| t18:1 c22:1 Cer | 0.15±0.05 | 0.05±0 | 0.05±0.01 | 0.08±0 |
| t18:1 c24:0 Cer | 1.88±0.26 | 1.24±0.11 | 3.93±2.24 | 1.72±0.14 |
| t18:1 c24:1 Cer | 1.43±0.17 | 1.74±0.2 | 1.58±0.47 | 1.7±0.22 |
| t18:1 c26:0 Cer | 12.67±1.53 | 14.93±1.29 | 16.78±2.03 | 17.46±1.83 |
| t18:1 c26:1 Cer | 1.11±0.15 | 0.8±0.18 | 1.38±0.69 | 0.82±0.03 |
| d18:0 h16:0 hCer | 1.77±0.2 | 1.17±0.09 | 1.18±0.13 | 1.38±0.1 |
| d18:0 h24:0 hCer | 0.16±0.02 | 0.16±0.01 | 0.51±0.37 | 0.17±0.02 |
| d18:1 h16:0 hCer | 0.55±0.06 | 0.47±0.1 | 0.51±0.09 | 0.51±0.04 |
| d18:1 h22:0 hCer | 0.08±0.03 | 0.06±0 | 0.09±0.01 | 0.08±0.02 |
| d18:1 h24:0 hCer | 2.31±0.42 | 2.2±0.25 | 2.2±0.33 | 2.42±0.26 |
| d18:1 h24:1 hCer | 0.12±0.04 | 0.14±0.02 | 0.1±0.02 | 0.12±0.01 |
| t18:0 h16:0 hCer | 5.02±1.2 | 1.67±0.13 | 2.13±0.24 | 2.13±0.28 |
| t18:0 h20:0 hCer | 0.15±0.04 | 0.17±0.03 | 0.22±0.02 | 0.29±0.04 |
| t18:0 h22:0 hCer | 2.06±0.29 | 1.71±0.32 | 2.11±0.14 | 3.39±0.32 |
| t18:0 h24:0 hCer | 1.36±0.06 | 1.03±0.07 | 1.62±0.22 | 1.89±0.23 |
| t18:0 h24:1 hCer | 6.11±2.14 | 6.21±0.71 | 4.19±1.19 | 4.77±1 |
| t18:0 h26:0 hCer | 3.62±0.49 | 3.37±0.5 | 4.13±0.79 | 5.68±1.19 |
| t18:1 h16:0 hCer | 7.59±1.16 | 4.83±0.7 | 6.01±0.3 | 7.47±0.84 |
| t18:1 h20:0 hCer | 0.77±0.11 | 0.59±0.03 | 0.63±0.03 | 1.06±0.17 |
| t18:1 h22:0 hCer | 4.64±0.21 | 4.16±0.01 | 4.25±0.31 | 6.03±0.65 |
| t18:1 h24:0 hCer | 33.93±2.33 | 30.72±2.62 | 32.92±1.11 | 42.46±5.09 |
| t18:1 h24:1 hCer | 9.25±2.35 | 8.75±0.37 | 7.26±1.62 | 11.76±0.22 |
| t18:1 h26:0 hCer | 20.88±2.21 | 17.59±1.41 | 17.72±1.11 | 26.12±2.88 |
| t18:1 h26:1 hCer | 4.56±0.47 | 3.33±0.38 | 4.02±0.2 | 4.56±0.38 |
| d18:0 h16:0 GlcCer | 0.52±0.15 | 0.63±0 | 0.45±0.18 | 0.59±0.01 |
| d18:1 h16:0 GlcCer | 128.59±6 | 100.9±5.31 | 98.73±9.89 | 104.11±7.43 |
| d18:1 h22:0 GlcCer | 0.23±0.04 | 0.22±0 | 0.21±0.01 | 0.17±0.01 |
| d18:1 h24:0 GlcCer | 0.47±0.05 | 0.32±0.04 | 0.22±0.07 | 0.32±0.04 |
| d18:1 h24:1 GlcCer | 2.76±0.08 | 2.49±0.06 | 2.39±0.24 | 2.21±0.15 |
| d18:1 h26:1 GlcCer | 0.08±0 | 0.06±0.01 | 0.1±0.02 | 0.09±0.01 |
| t18:0 h24:0 GlcCer | 0.21±0.02 | 0.16±0.01 | 0.16±0.01 | 0.19±0.01 |
| t18:1 h16:0 GlcCer | 22.23±0.7 | 17.92±0.55 | 18.27±0.19 | 21.21±1.1 |
| t18:1 h18:0 GlcCer | 0.25±0.01 | 0.16±0.01 | 0.15±0 | 0.19±0.02 |
| t18:1 h20:0 GlcCer | 0.41±0.03 | 0.26±0.01 | 0.27±0.01 | 0.34±0.04 |
| t18:1 h22:0 GlcCer | 10.04±0.93 | 9.19±0.4 | 7.01±1.15 | 8.61±0.15 |
| t18:1 h22:1 GlcCer | 0.32±0.02 | 0.27±0.01 | 0.25±0.03 | 0.3±0.03 |
| t18:1 h24:0 GlcCer | 24.34±0.75 | 20.02±0.24 | 17.31±1.71 | 21±1.43 |
| t18:1 h24:1 GlcCer | 38.01±9.82 | 40.92±0.11 | 28.12±9.25 | 38.68±0.53 |
| t18:1 h26:0 GlcCer | 3.08±0.69 | 2.07±0.16 | 2.02±0.35 | 2.43±0.04 |
| t18:1 h26:1 GlcCer | 3.88±0.52 | 3.51±0.04 | 2.73±0.48 | 3.33±0.18 |
| d18:1 h16:0 GIPC | 23.1±3.05 | 27.54±1.86 | 20.88±1.56 | 30.17±4.61 |
| d18:0 h16:0 GIPC | 24.42±2.8 | 14.79±0.4 | 13.43±1.31 | 21.14±1.8 |
| t18:1 h16:0 GIPC | 23.7±0.97 | 23.11±1.7 | 20.35±0.21 | 28.54±1.99 |
| t18:0 h16:0 GIPC | 17.36±2.99 | 7.57±0.54 | 7.28±0.99 | 9.17±1.11 |
| t18:1 h20:0 GIPC | 0.94±0.32 | 1.1±0.29 | 0.95±0.28 | 1.24±0.17 |
| d18:0 h22:0 GIPC | 0.47±0.14 | 1.02±0.3 | 1.31±0.28 | 1.62±0.66 |
| t18:1 h22:0 GIPC | 13.2±1.79 | 15.73±0.71 | 13.4±0.87 | 19.48±1.86 |
| t18:0 h22:0 GIPC | 2.78±0.49 | 4.61±0.15 | 3.6±0.56 | 5.32±1.08 |
| d18:0 h24:1 GIPC | 0.36±0.12 | 0.52±0.11 | 0.59±0.27 | 1.43±0.54 |
| d18:0 h24:0 GIPC | 0.38±0.22 | 0.6±0.03 | 0.54±0.19 | 1.21±0.26 |
| t18:1 h24:1 GIPC | 29.27±3.62 | 34.19±0.75 | 30.96±2.32 | 38.81±4.06 |
| t18:1 h24:0 GIPC | 25.51±4.04 | 33.99±1.6 | 30.98±3.52 | 40.24±6.5 |
| t18:0 h24:1 GIPC | 10.55±2.28 | 12.6±3.56 | 12.59±1.42 | 12.08±3.7 |
| t18:0 h24:0 GIPC | 9.7±1.36 | 10.72±1.29 | 11.2±1.31 | 15.38±2.92 |
| d18:0 h26:1 GIPC | 0.42±0.17 | 0.44±0.23 | 0.28±0.11 | 0.09±0.05 |
| d18:0 h26:0 GIPC | 0.29±0.1 | 0.34±0.19 | 0.13±0.06 | 0.29±0.13 |
| t18:1 h26:1 GIPC | 3.35±0.47 | 4.39±0.34 | 4.09±0.63 | 5.33±1.04 |
| t18:1 h26:0 GIPC | 7.1±1.05 | 10.28±0.7 | 9.55±1.53 | 14.75±2.66 |
| t18:0 h26:1 GIPC | 10.56±1.48 | 15.66±1.29 | 14.75±2.11 | 22.45±4.52 |
| t18:0 h26:0 GIPC | 1.68±1.05 | 1.19±0.37 | 0.85±0.59 | 2.67±1.81 |

**Supplemental Table 6.** Sequence of primers used in this study.

| **Gene** | **Primer** | **Sequence (5’→3’)** | **Usage** |
| --- | --- | --- | --- |
| *ACT2* | Froward | GGTAACATTGTGCTCAGTGGTGG | qRT-PCR |
|  | Reverse | GGTGCAACGACCTTAATCTTCAT |  |
| *EDS1* | Froward | AGCTTCTGTGGAAATGGCTGTGAGG | qRT-PCR |
|  | Reverse | TGTCACACAACGAGGCTCAAGGTA |  |
| *PAD4* | Froward | TGTGGGCTTGCCAGTCACCG | qRT-PCR |
|  | Reverse | GTGCGGTGAAAGCGGCCAAT |  |
| *SID2* | Froward | AGTTCTGTCTTCAACCACCTG | qRT-PCR |
|  | Reverse | TCTATCTCCATATCACGAGCACTA |  |
| *FMO1* | Froward | ACAATGGTGGTGAGAACAACT | qRT-PCR |
|  | Reverse | CGAAGAGTAGAACAAGAAGAATGGT |  |
| *PR1* | Froward | GTGAGGTGTAACAATGGTGGAA | qRT-PCR |
|  | Reverse | CTTCATTAGTATGGCTTCTCGTTCA |  |
| *SAG13* | Froward | GATATTTATGCTTGCGTGGTGAAC | qRT-PCR |
|  | Reverse | CTAGTCTGCCGTCAAATTGGTAA |  |
| *LCB2b* | Froward | GGCTCACCTTTACGCCACA | qRT-PCR |
|  | Reverse | CTCCAAGAACTTCAAACCCCA |  |
| *LOH1* | Froward | TCCGATTCTGAAAGCGATGATG | qRT-PCR |
|  | Reverse | ATTCCTAGTCTCCGTGTGGTT |  |
| *LOH2* | Froward | GGATTCTTCTTCTTGAGGCTTGTC | qRT-PCR |
|  | Reverse | CCGAGTAGCAGCATCATTCAAT |  |
| *LOH3* | Froward | CTCTCCTATATTGCTTGCTTGTTCT | qRT-PCR |
|  | Reverse | AATCAGTCTTCGTGCTCATCTTC |  |
| *EDS1* | Froward | GAAGAAGCAGGAGCAGTCGTAATC | *eds1-2* mutant identification |
|  | Reverse | CGATTTGTGATTTTTGGGAAGCGT |  |
| *PAD4* | Froward | ACAAGAATTGTTCGGGCTCCT | *pad4-1* mutant identification |
|  | Reverse | GCTTGCTCAAGCTTAGCCCAA |  |
| *SID2* | Froward | CAACCACCTGGTGCACCAGC | *sid2-1* mutant identification |
|  | Reverse | AAGCAAAATGTTTGAGTCAGCA |  |
