# Supplementary material for: The Two Classes of Ceramide Synthases Play Different Roles in Plant Immunity and Cell Death
Source: Front Plant Sci. 2022 Apr 7;13:824585. doi: 10.3389/fpls.2022.824585 (PMC9021646; doi:10.3389/fpls.2022.824585)
Supplement: Supplementary file 2 [file Data_Sheet_2.docx]

**Supplemental data**


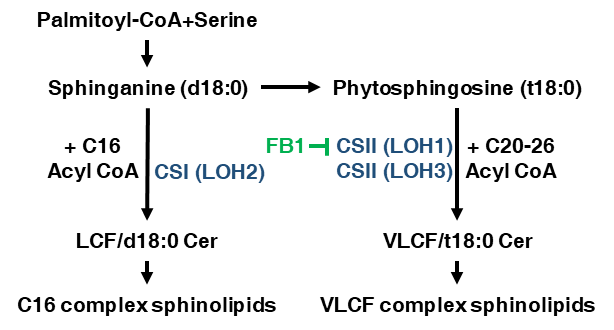


**SUPPLEMENTARY FIGURE 1.** *De novo* Cer biosynthesis mediated by CSs in Arabidopsis.

CS: ceramide synthase; LOH: LAG1 HOMOLOG; FB1: Fumonisin B1; LCF: long-chain fatty acid; VLCF: very-long-chain fatty acid.


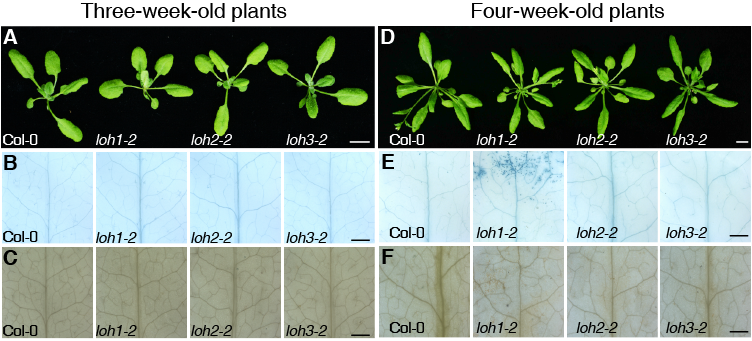


**SUPPLEMENTARY FIGURE 2.** Phenotype of 3- and 4-week-old Col-0, *loh1-2*, *loh2-1*, *loh3-2* plants.

**(A)** Representative images of 3-week-old plants. Bar = 1 cm.

**(B)** Trypan blue staining of leaves from **(A)**. Bar = 1 mm.

**(C)** DAB staining of leaves from **(A)**. Bar = 1 mm.

**(D)** Representative images of 4-week-old plants. Bar = 1 cm.

**(E)** Trypan blue staining of leaves from **(D)**. Bar = 1 mm.

**(F)** DAB staining of leaves from **(D).** Bar = 1 mm.

At least 30 plants per line were tested each time in **(A, D)**, and at least 12 leaves per line were stained each time in **(B, C, E, F)**. The experiments were conducted three times independently.


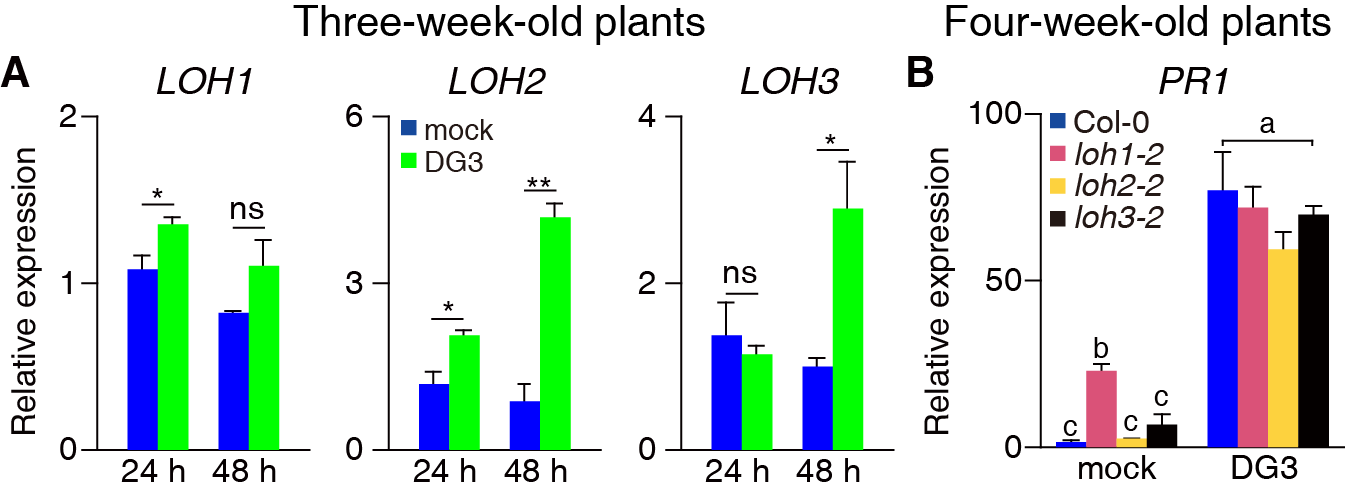


**SUPPLEMENTARY FIGURE 3.** The role of LOHs in plant immunity.

**(A)** Relative transcript levels of *LOH1*, *LOH2*, and *LOH3* in 3-week-old Col-0 plants 24 or 48 h post *Psm*DG3 infection.

**(B)** Relative *PR1* transcript levels in 4-week-old Col-0, *loh1-2*, *loh2-2*, and *loh3-2* plants 24 or 48 h after *Psm*DG3 inoculation.

*ACT2* served as internal control. Values were normalized to Col-0 levels (set to 1). Values are means ± SE from triplicate biological repeats. Significant differences between the mock and *Psm*DG3, were determined by Student’s t-test (**P* < 0.05, ***P* < 0.01, and ns indicates no significance difference) in **(A)**. Different letters indicate significant differences between all genotypes/treatments, as determined by Fisher’s PLSD (*P* < 0.05) in **(B).** All experiments were conducted at least three times independently.


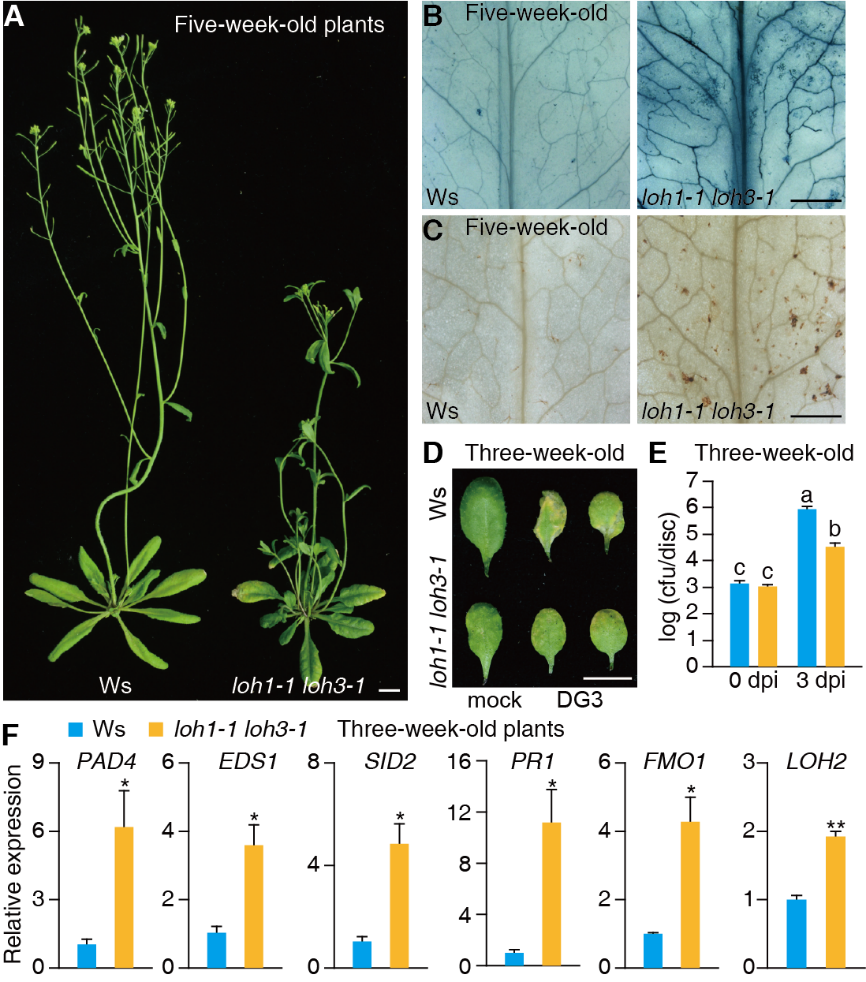


**SUPPLEMENTARY FIGURE 4.** Defense responses of *loh1-1 loh3-1*.

**(A)** Representative images of 5-week-old plants. Bar = 1 cm.

**(B)** Trypan blue staining of leaves from **(A)**. Bar = 1 mm.

**(C)** DAB staining of leaves from **(A)**. Bar = 1 mm.

**(D)** Representative images of 3-week-old plants 3 d after inoculation with *Psm*DG3.

**(E)** Growth of *Psm*DG3 in plants from **(D)**.

**(F)** Relative transcript levels of *PAD4*, *EDS1*, *SID2*, *PR1*, *FMO1*, and *LOH2* in 3-week-old plants. *ACT2* served as the internal control. All values were normalized to Ws (set to 1).

At least 30 plants per line were tested each time in **(A, D, E)**, at least 12 leaves per line were stained each time in **(B, C)**. Values are means ± SE from six biological replicates at 0 dpi and twenty-four biological replicates at 3 dpi in **(E)**, and triplicate biological replicates in **(F)**. Different letters indicate significant differences between all genotypes/time points, as determined by Fisher’s PLSD (*P* < 0.05) in **(E)**. Significant differences between the Ws and *loh1-1 loh3-1* were determined by Student’s t-test (**P* < 0.05, ***P* < 0.01) in **(F)**. All experiments were conducted at least three times using independent samples.


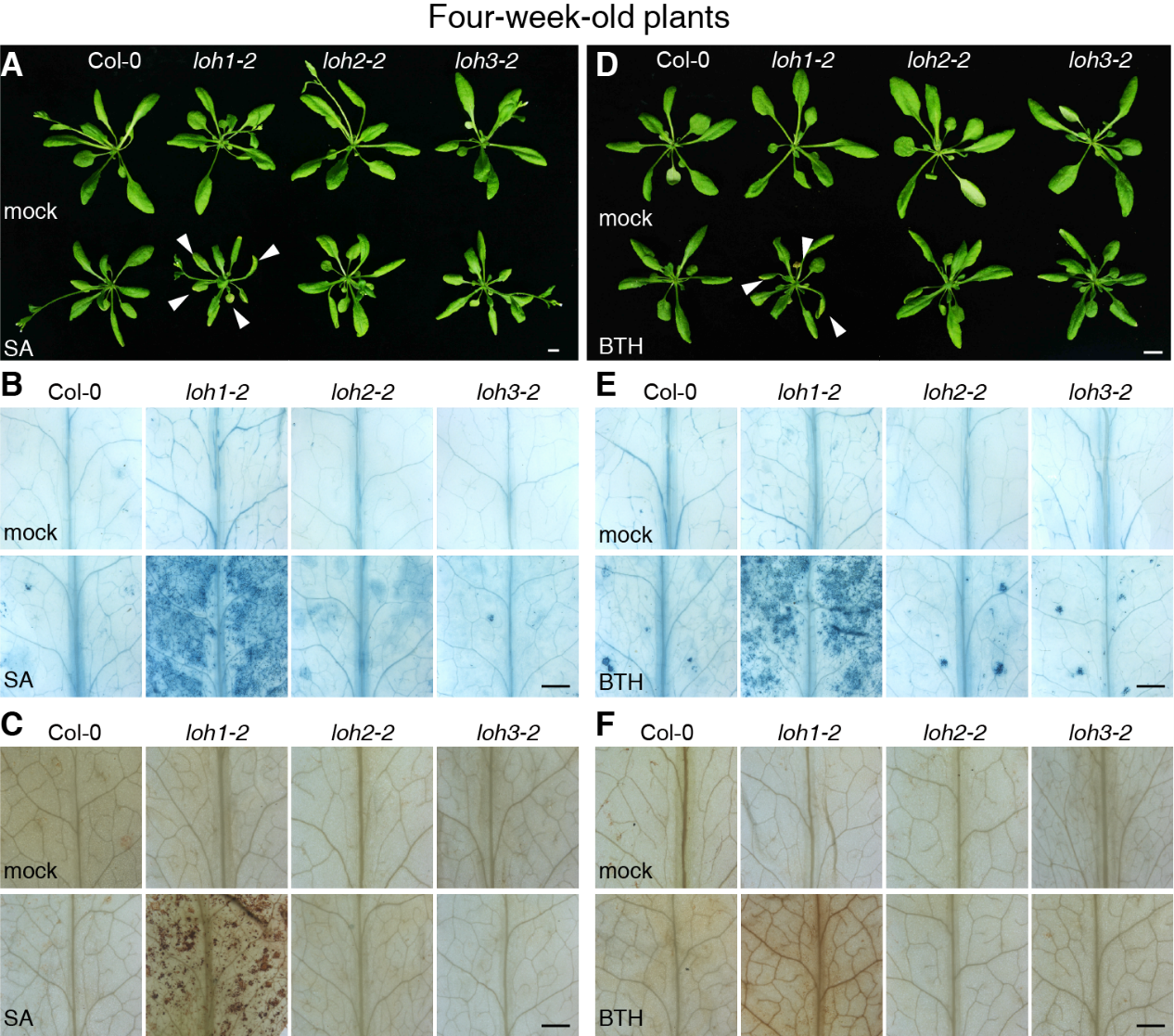


**SUPPLEMENTARY FIGURE 5.** Effect of *pad4-1*, *eds1-2*, and *sid2-1* mutant alleles on SA- or BTH-induced cell death in the *loh1-2* mutant.

Three-week-old Col-0, *loh1-2*, *loh2-1*, and *loh3-2* plants were treated with 200 μM SA or 0.1% ethanol (mock), 300 μM BTH or 0.3% acetone (mock).

**(A)** Representative images of 3-week-old plants 1 week post SA treatment. Bar = 1 cm.

**(B)** Trypan blue staining of leaves from **(A)**. Bar = 1 mm.

**(C)** DAB staining of leaves from **(A)**. Bar = 1 mm.

**(D)** Representative images of 3-week-old plants 1 week post BTH treatment. Bar = 1 cm.

**(E)** Trypan blue staining of leaves from **(D)**. Bar = 1 mm.

**(F)** DAB staining of leaves from **(D)**. Bar = 1 mm.

At least 30 plants per line were tested each time in **(A, D)**, and at least 12 leaves per line were stained each time in **(B, C, E, F)**. All experiments were conducted at least three times using independent samples.


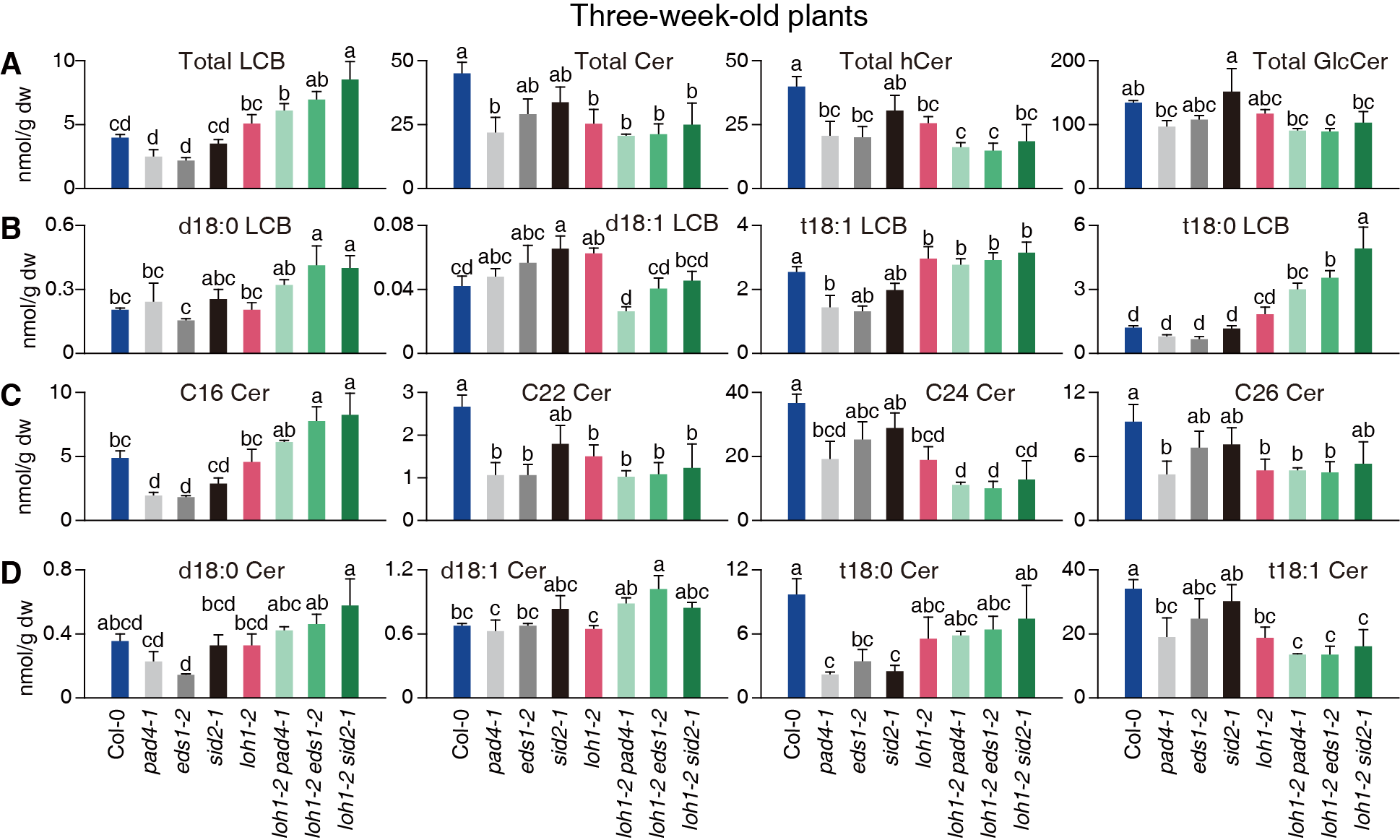


**SUPPLEMENTARY FIGURE 6.** Sphingolipid profile of 3-week-old Col-0, *pad4-1*, *eds1-2*, *sid2-1*, *loh1-2 pad4-1*, *loh1-2 eds1-2*, and *loh1-2 eds1-2* plants.

**(A)** Total contents for LCB, Cer, hCer and GlcCer.

**(B)** Contents of d18:0, d18:1, t18:0, and t18:1 LCB.

**(C)** Contents of Cer species with FA moieties.

**(D)** Contents of Cer species with LCB moieties.

Values are means ± SE from triplicate biological repeats. Different letters indicate significant differences between genotypes, as determined by Fisher’s PLSD (*P* < 0.05). The experiments were conducted three times independently.


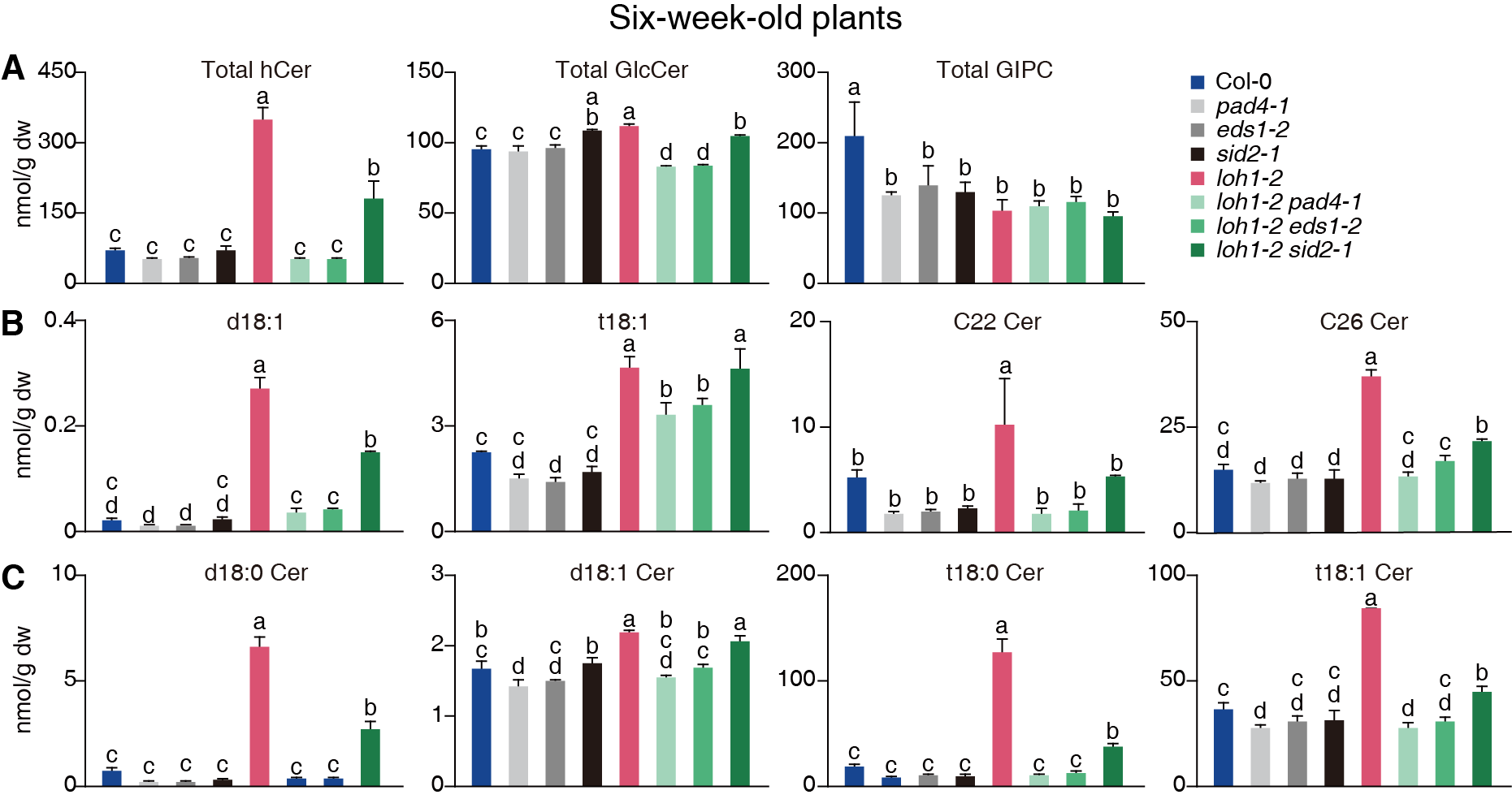


**SUPPLEMENTARY FIGURE 7.** Sphingolipid profiles of 6-week-old Col-0, *pad4-1*, *eds1-2*, *sid2-1*, *loh1-2 pad4-1*, *loh1-2 eds1-2*, and *loh1-2 sid2-1* plants.

**(A)** Contents of total hCer, GlcCer and GIPC.

**(B)** Contents of d18:1, t18:1, C22 Cer and C26 Cer.

**(C)** Contents of Cer species with LCB moieties.

Values are means ± SE from triplicate biological repeats. Different letters indicate significant differences between genotypes, as determined by Fisher’s PLSD (*P* < 0.05). The experiments were conducted three times independently.


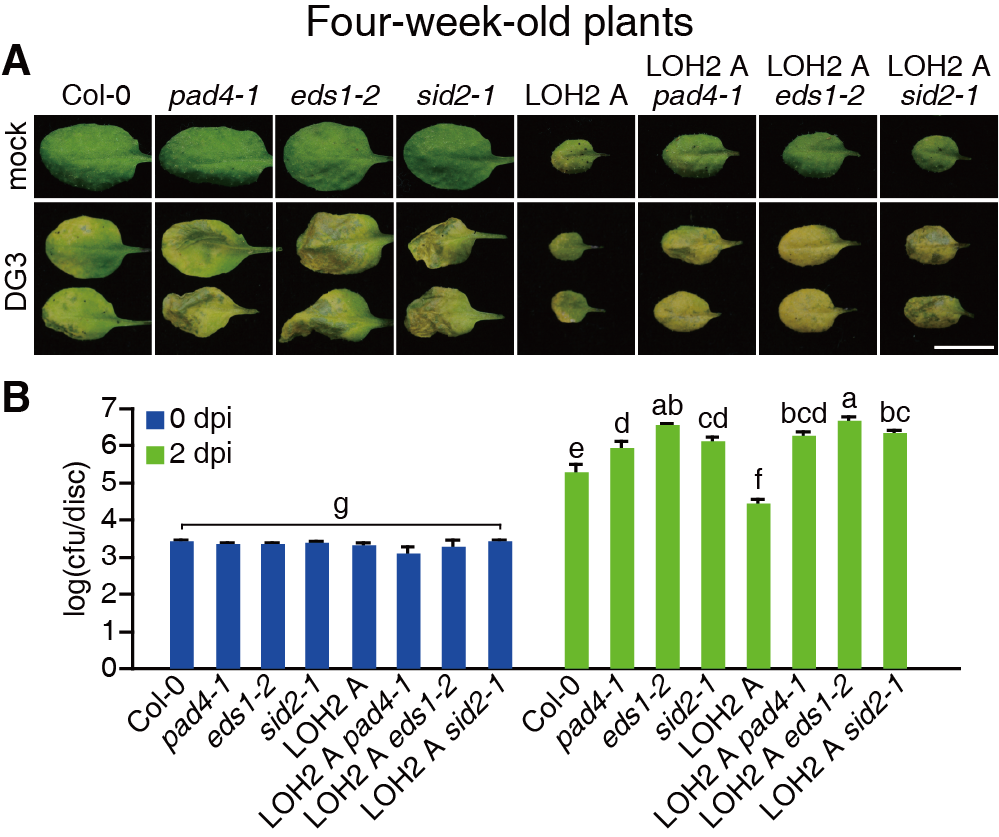


**SUPPLEMENTARY FIGURE 8.** Responses of *LOH2-*overexpression lines to *Psm*DG3 infection.

**(A)** Representative images of the leaves from 4-week-old plants 2 d after *Psm*DG3 inoculation. Bar = 1 cm.

**(B)** Growth of *Psm*DG3 in plants from **(A)**.

30 plants were used in **(A, B)**. Values are means ± SE from six biological replicates at 0 dpi and twenty-four biological replicates at 2 dpi in **(B)**. Significant differences between all genotypes/time points were determined by Fisher’s PLSD (*P* < 0.05) in **(B)**. The experiments were conducted three times independently.


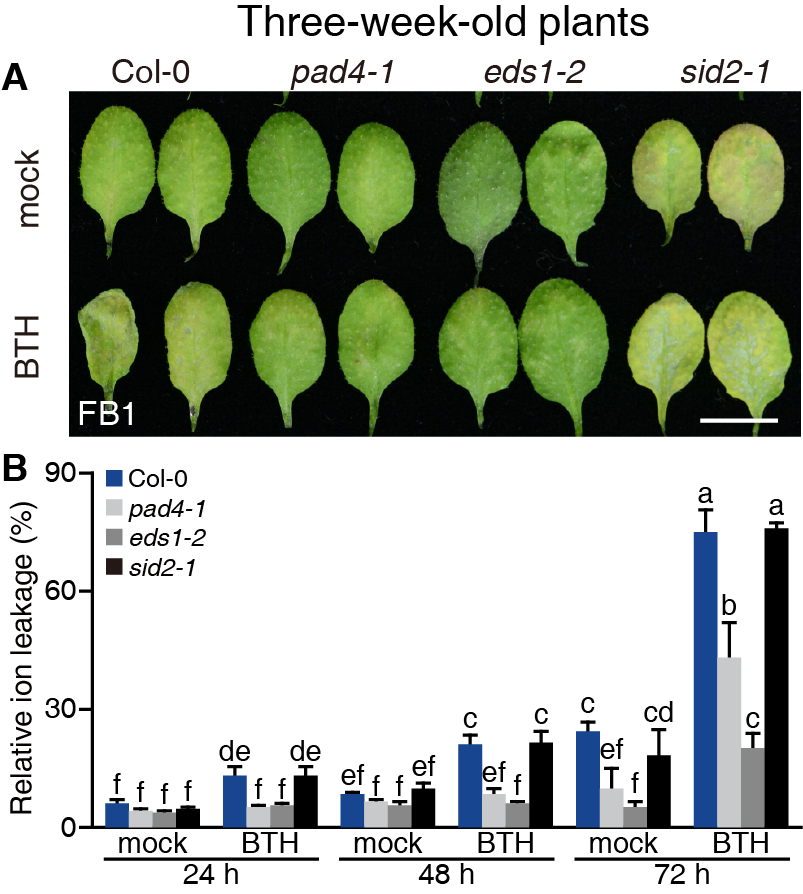


**SUPPLEMENTARY FIGURE 9.** Responses of *pad4-1*, *eds1-2*, and *sid2-1* to combined treatment with BTH and FB1.

**(A)** Representative images of leaves 4 d after treatment. Three-week-old plants were sprayed with mock or 300 μM BTH, and infiltrated with 10 μM FB1 1 day later. Bar = 1 cm.

**(B)** Relative ion leakage in plants 24, 48, and 72 h after treatments.

At least 30 plants per line were tested each time in **(A, B)**. Values are means ± SE from triplicate biological repeats. Different letters indicate significant differences between all genotypes/time points/treatments, as determined by Fisher’s PLSD (*P* < 0.05). Significant differences. All experiments were conducted at least three times using independent samples.


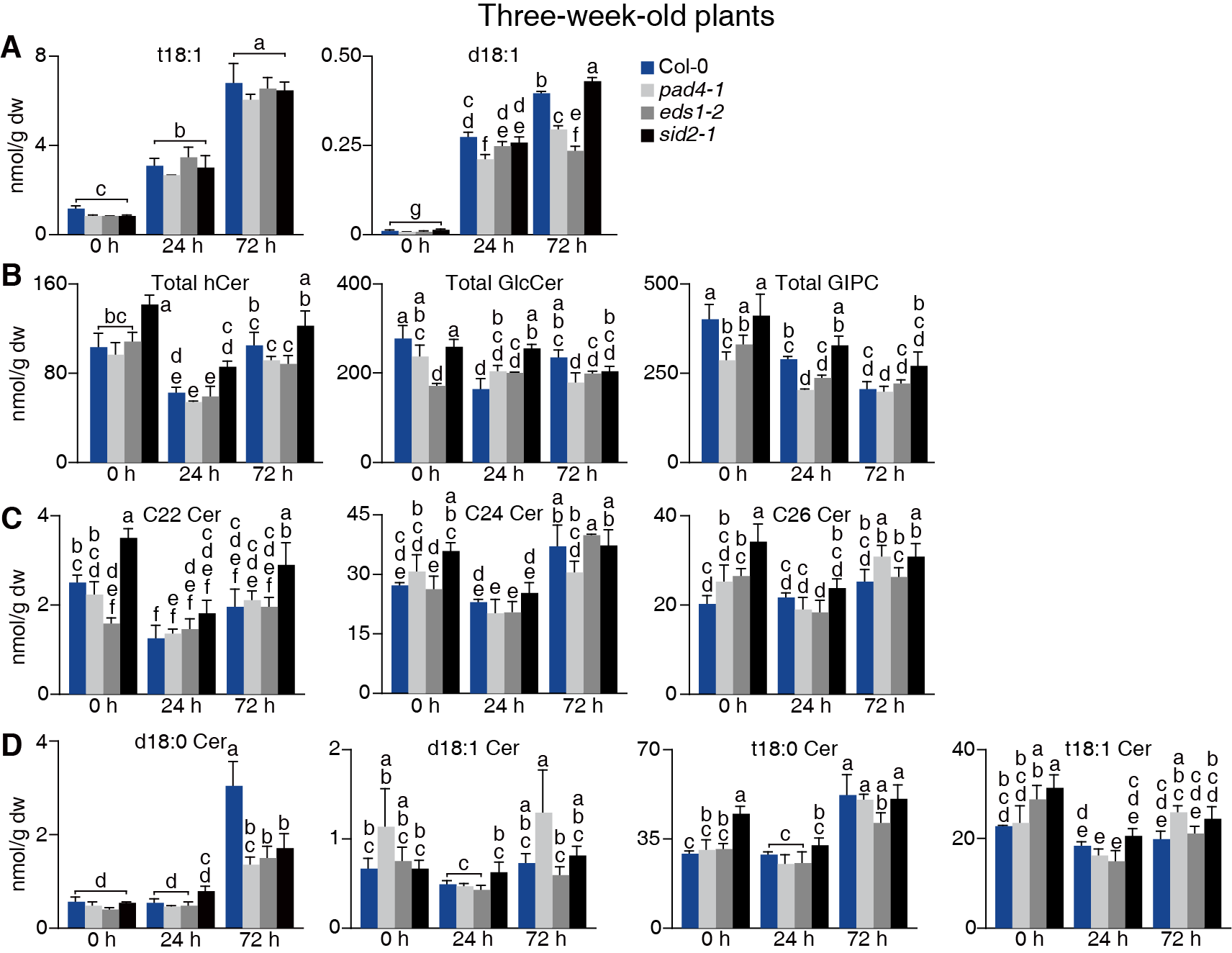


**SUPPLEMENTARY FIGURE 10.** Sphingolipid profiles of Col-0, *pad4-1*, *eds1-2*, and *sid2-1* plants after FB1 treatment*.*

**(A)** Contents for t18:1 and d18:1.

**(B)** Total contents of hCer, GlcCer and GIPC.

**(C)** Contents of C22, C24, and C26 Cer.

**(D)** Content of Cer species with LCB moieties.

Three-week-old plants were treated with mock or 10 μM FB1 for 24 or 72 h. Values are means ± SE from triplicate biological repeats. Different letters indicate significant differences between all genotypes/time points, as determined by Fisher’s PLSD (*P* < 0.05). The experiments were conducted three times independently.


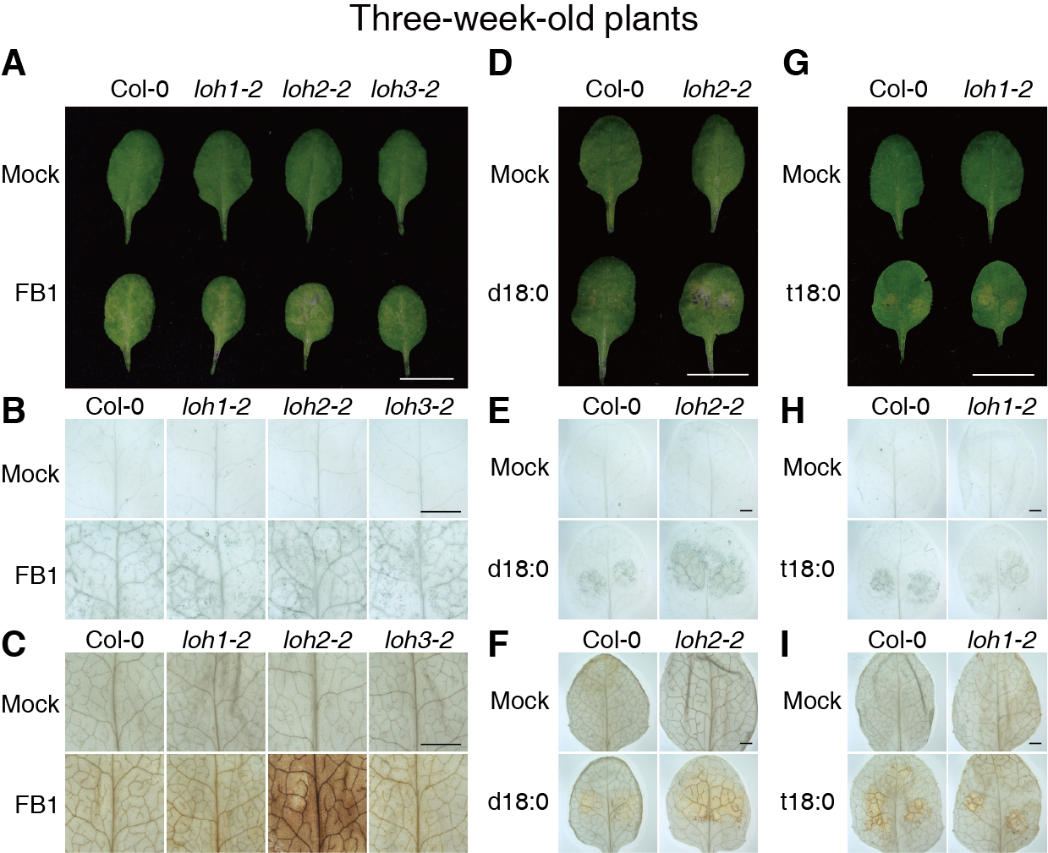


**SUPPLEMENTARY FIGURE 11.** Responses of wild type and *lohs* to FB1 or LCB treatment.

**(A)** Representative images of leaves 60 h post treatment. Three-week-old Col-0, *loh1-2*, *loh2-2* and *loh3-2* plants treated with mock or 10 μM FB1. Bar = 1 cm.

**(B)** Trypan blue staining of leaves from **(A)**. Bar = 1 mm.

**(C)** DAB staining of leaves from **(A)**. Bar = 1 mm.

**(D)** Representative images of leaves 60 h post treatment. Three-week-old Col-0, and *loh2-2* plants treated with mock or 1 mM d18:0. Bar = 1 cm.

**(E)** Trypan blue staining of leaves from **(D)**. Bar = 1 mm.

**(F)** DAB staining of leaves from **(D)**. Bar = 1 mm.

**(G)** Representative images of leaves 60 h post treatment. Three-week-old Col-0, and *loh1-2* plants treated with mock or 1 mM t18:0. Bar = 1 cm.

**(H)** Trypan blue staining of leaves from **(G)**. Bar = 1 mm.

**(I)** DAB staining of leaves from **(G)**. Bar = 1 mm.

At least 30 plants per line were tested each time in **(A, D, G)** and at least 12 leaves per line were stained each time in **(B, C, E, F, H, I)**. All experiments were conducted at least three times using independent samples.
